# Direct Electrochemical Detection of Geosmin Using Zn_2_Ti_3_O_8_ Nanosheets Grown on Graphene Oxide

**DOI:** 10.1021/acsomega.6c02052

**Published:** 2026-06-10

**Authors:** Nádia Cristina da Silva Iack, Kelly Leite dos Santos Castro Assis, Warley Cirqueira Machado, Druval Santos de Sá, Thayane Almeida de Medeiros, Carolina Carvalho de Mello, Maybi Falker Sampaio, Maria Luiza de Araujo Dorneles, Fernando Stavale, Adriana Maria da Silva, Braulio Soares Archanjo, Carlos Alberto Achete

**Affiliations:** † Postgraduate Metrology and Technology Program, National Institute of Metrology, Quality and Technology, 25250-020 Duque de Caxias, RJ, Brazil; ‡ Materials Division, National Institute of Metrology, Quality and Technology, 25250-020 Duque de Caxias, RJ, Brazil; § Brazilian Center for Research in Physics, 22290-180 Rio de Janeiro, RJ, Brazil

## Abstract

The direct electrochemical detection of geosmin, a pervasive odor-causing compound in water, poses a longstanding analytical challenge owing to its exceptionally high oxidation potential. Here, we introduce a groundbreaking sensing architecture that achieves unprecedented direct anodic oxidation of geosmin using ultrathin, defective Zn_2_Ti_3_O_8_ spinel nanosheets synthesized via a simple graphene oxide (GO)-confined growth. This innovative confined approach unexpectedly stabilizes a defective Zn_2_Ti_3_O_8_ phase in a two-dimensional (2D) morphology, enabling superior charge transport and robust electrode–solution interfacial interactions. Integrated onto a glassy carbon electrode (GCE), the resulting platform dramatically lowers the overpotential for geosmin oxidation, circumventing the intrinsic limitations of conventional electrochemical sensors. Under optimized voltammetric conditions, the sensor delivers a linear dynamic range of 0.36–3.6 μg mL^–1^, with limits of detection (LOD) and quantification (LOQ) of 0.08 and 0.27 μg mL^–1^, respectively. The device exhibits excellent reproducibility, satisfactory recovery in spiked samples, and robust tolerance to common interferents. Beyond superior analytical metrics, this novel material establishes a simplified electrochemical approach for direct electrochemical monitoring of odorants in aqueous matrices, opening promising avenues for the design of affordable, portable sensors for real-time water quality assessment in field settings.

## Graphical abstract

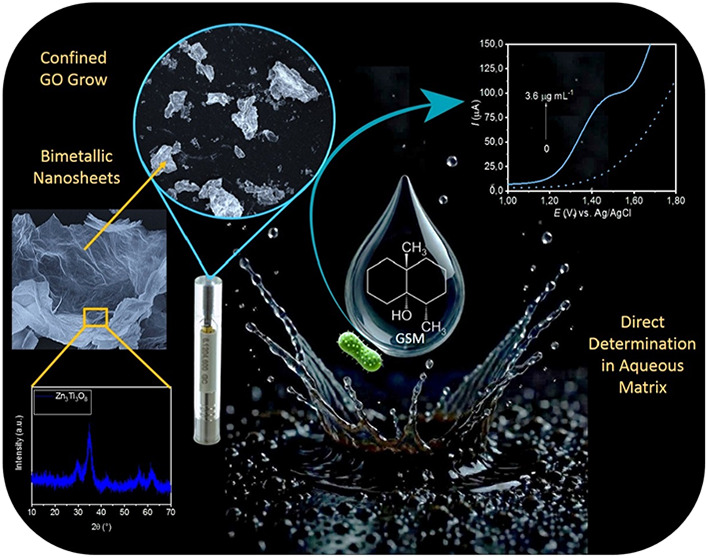

## 1 Introduction

Water pollution is one of the most pressing challenges of our time, driving a growing body of research into the efficacy, quality, and safety of treatments used in water treatment facilities. Certain compounds are notorious for imparting objectionable odor in water, notably geosmin (GSM), which produces a characteristic earthy odor and musty taste derived from some species of fungi and blue-green algae.^1–3^ GSM is widely distributed in aquatic environments. Owing to its earthy odor, even trace levels (<10 ng L^–1^) in drinking water can render it sensorially unacceptable. The human olfactory detection threshold for GSM typically ranges from 1 to 10 ng L^–1^.^1,4^ In natural waters, GSM concentrations are typically found at low ng L^–1^ levels; however, during algal bloom events or microbial proliferation, these concentrations can increase significantly, reaching tens to hundreds of ng L^–1^ and, in extreme cases, even μg L^–1^ levels.^1–8^ These episodic increases pose a major challenge for water treatment facilities, as conventional processes are often insufficient for complete removal. Taste and odor issues in drinking water not only impair potability and perceived quality but also lead to considerable economic losses in the aquaculture industry.^5^ Furthermore, mutagenicity and hepatotoxicity caused by GSM have been reported in some studies.^6,7^ Although the toxicity of GSM to humans has not yet been demonstrated, a recent study using zebrafish embryos as a model investigated the biological effects of environmentally relevant concentrations (50, 500, 5000 ng L^–1^) of GSM on developing zebrafish.^8^ The findings revealed that GSM can simultaneously triggers oxidative stress, apoptosis, and endocrine disruption. A more recent research from the same group demonstrated that GSM disrupts energy metabolism and locomotor behavior in zebrafish during early life stages.^9^ Although GSM is widely recognized as nontoxic and affects only the sensory quality of drinking water, additional long-term studies at very high concentrations could further confirm its safety profile. Consequently, rapid and effective detection of GSM in water is essential to guarantee the aesthetic quality and sustain consumer trust in drinking water safety. Despite its significant impact on water quality, the reliable analytical determination of GSM at low concentration levels remains challenging, particularly for rapid and decentralized monitoring applications.

The detection of GSM is commonly performed using stablished analytical techniques such as chromatography (GC), gas chromatography–mass spectrometry (GC–MS), and chemiluminescence.^1,3,5,10^ Although these methods offer high sensitivity, they are time-consuming and require extensive sample preparation, sophisticated instrumentation, and skilled operators, thereby restricting their use for routine monitoring or on-site analysis. Consequently, sample throughput remains limited, compromising the statistical robustness of monitoring campaigns across large water bodies or the distribution network. Thus, there is an urgent need for highly sensitive, rapid techniques capable of detecting GSM at traces levels (ng/L range). Electrochemical sensors hold considerable promise in this context, as they have the potential to match or superpass traditional chromatographic systems while providing key advances, such as portability, low cost, minimal sample pretreatment, ease of operation, and real-time results.^11,12^ Despite these benefits, electrochemical approaches for GSM detection remain limited due to the high GSM oxidation potential at practical electrode potentials,^4^ which hinders direct anodic oxidation. This challenge underscores the critical need for innovative electrochemical sensors capable of achieving sufficient low limits of detection (LOD) through material-enabled strategies that overcome conventional overpotential barriers. Recent advances in electrochemical sensing have demonstrated that nanostructured materials, particularly defect-engineered metal oxides and hybrid composites, can significantly enhance sensitivity, selectivity, and charge transfer properties.^13–15^ In this context, the design of nanoscale architectures with high surface area and abundant active sites has emerged as an effective strategy for improving sensor performance.

Several studies reported in the literature employ enzymatic systems, molecularly imprinted polymers (MIPs), or indirect detection strategies for the determination of GSM. Although effective, these approaches introduce additional complexity to the analytical procedure. For instance, Braga and collaborators developed an electronic tongue based on nonspecific polymeric sensors and impedance measurements, enabling the monitoring and quantification of GSM in water samples with an LOD of 25 ng L^–1^.^10^ Similarly, a bioelectronic nose integrating the human olfactory receptor (hOR) with a single-walled carbon nanotube field-effect transistor (SWCNT-FET) has been reported, achieving selective detection of both GSM and 2-methylisoborneol (2-MIB) at concentrations as low as 10 ng L^–1^.^16^ Another interesting approach relies on the combination of high-performance liquid chromatography (HPLC) with electrochemical detection by using a platinum nanoparticle-embedded nanocarbon film electrode, achieving an LOD of 100 ng L^–1^ for GSM.^14^ Li et al. developed a method for the detection of GSM and MIB using a competitive displacement technique based on MIPs and fluorescent tags for the detection of GSM and MIB. The LOD of GSM was 69,000 ng L^–1^ for GSM.^14^ Researchers have also developed an electrochemical sensor by modifying a glassy carbon (GC) electrode surface through an MIP-based electrochemical sensor by modifying a GC electrode for GSM detection via signal attenuation. In this approach, GSM binding to the imprinting cavities blocks the redox provide access, resulting in a decrease in current that is proportional to the analyte concentration. This sensor achieved a remarkable LOD of 5 ng L^–1^.^1^ However, despite the advantages of electrochemical sensors, the direct electrochemical detection of GSM is inherently challenging due to its high oxidation potential and weak electroactivity on conventional electrode surfaces.

Despite recent advances in GSM monitoring, many methodologies reported in the literature over the last five years continue to face limitations, including an intricate sensor electrode fabrication process, elevated costs, and limits of detection (LODs) that often exceed environmentally relevant thresholds, including the human detection olfactory limit in the range of 4–10 ng·L^–1^.^1,13,14^ For instance, while molecularly imprinted polymer (MIP)-based electrochemical sensors have achieved outstanding LODs such as 5 ng·L^–1^ through indirect signal attenuation^1^ and bioelectronic noses with field-effect transistors have reached 10 ng·L^–1^ via olfactory receptor integration,^13^ these approaches often require time-consuming laboratory work by bioprobe immobilization or hybrid chromatographic protocols, affecting the on-site applications while raising costs.^10,14^ Thus, demonstrating direct electrochemical detection of GSM overcomes the mentioned limitations, opening new possibilities to GSM monitoring in water by enabling rapid and in-field assessments. In this regard, the development of an electrochemical sensor for direct GSM detection can add substantial analytical advancement, delivering a distinctive blend of operational simplicity, affordability, and enhanced sensitivity, addressing key shortcomings of existing methodologies. The direct electrochemical detection of GSM is a potential technology in order to facilitate real-time analysis in complex aqueous matrices. Moreover, the feasibility of applying directly to environmental samples minimizes pretreatment requirements while supporting continuous monitoring of water treatment facilities or portable field evaluations. Such a sensor would represent a pivotal breakthrough, markedly improving the monitoring efficiency and enabling prompt remedial measures in distribution systems. For this end, glassy carbon electrodes (GCEs) have emerged as a versatile platform for advancing direct electrochemical detection of GSM in water samples, as evidenced by recent studies.^15–19^ GCEs are favored for their exceptional attributes, including electrochemical inertness, chemical stability, superior electrical conductivity, impermeability, high hardness, ease of surface modification, and excellent reproducibility. Another outstanding advantage is related to costs in view of the material being economical with a broad potential window; GCE allows robust sensor architectures, promising candidates for overcoming the high oxidation overpotentials of GSM.^20–22^

Aiming to achieve high performance in the direct electrochemical detection of GSM, the GCE surface often requires modification to enhance sensitivity and selectivity, as reported in the literature.^15,16,18,19,23–25^ In this context, nanotechnology-based strategies have been widely explored to develop robust and efficient materials for electroanalysis. Among the various modification approaches, metal oxide nanosheets stand out as an excellent choice due to their unique physical, chemical, optical, and electronic properties at the nanoscale. Compared to their bulk counterparts, these nanomaterials exhibit superior electrical and photocatalytic performance, attributed to their high surface area, tunable size and morphology, and excellent stability.^17,18^ The ultrathin two-dimensional (2D) structure provides a high surface-to-volume ratio and abundant exposed active sites, further enhancing electrochemical performance.^19–22^ The effectiveness of 2D architectures in improving material performance has been demonstrated in various applications.^21,23–30^ Recently, we reported the outstanding performance of Nb_2_O_5_ nanosheets as a sensing platform for ibuprofen detection, achieving a low limit of detection (0.02 μM), high selectivity, and remarkable stability.^28^ These results highlight the great potential of 2D metal oxide nanomaterials for electrochemical sensing, while also emphasizing the need to develop new materials with optimized properties.

Conventionally, semiconducting metal oxides such as titanium dioxide (TiO_2_) and zinc oxide (ZnO) have been widely employed in photocatalysis, electrochemistry, and electrocatalysis owing to their suitable band gap, high chemical stability, abundance, low cost, and minimal environmental toxicity.^30–37^ The high specific surface area, fast electron transfer kinetics, and abundance of exposed active sites make TiO_2_ nanostructures highly promising for electrochemical applications. Similarly, ZnO stands out because of its large specific surface area, high adsorption capacity, high catalytic efficiency, excellent electron mobility, and remarkable chemical and thermal stability, making it particularly suitable for the electrochemical detection of various analytes. Furthermore, combining different nanostructured metal oxides often produces a pronounced synergistic effect, leading to significantly higher electrocatalytic activity than that of the corresponding single metal oxides. In particular, the integration of TiO_2_ and ZnO semiconductors gives rise to zinc titanates, an important class of materials that can exist in three polymorphic forms: ZnTiO_3_ (zinc metatitanate), Zn_2_TiO_4_ (zinc orthotitanate), and Zn_2_Ti_3_O_8_ (metastable zinc titanate).^38–41^ Among these, Zn_2_Ti_3_O_8_ has attracted increasing attention due to its high surface area, favorable electronic structure, and large band gap, making it suitable for electrochemical and photocatalytic applications.^42–44^ Despite these advantages, the controlled synthesis of ultrathin metal oxide nanosheets remains challenging. Among the available strategies, template-assisted self-assembly has emerged as an effective approach, in which the growth of metal oxides is directed by a planar structure.^45,46^ In this context, graphene oxide (GO) has been widely employed as a sacrificial template because of its high surface area and abundance of oxygen-containing functional groups, which enable strong interactions with metal precursors. Previous studies have demonstrated the successful synthesis of various metal oxides, including Nb_2_O_5_, TiO_2_, Fe_2_O_3_, SnO_2_, and NiO, using GO as a template, followed by its removal during calcination to yield ultrathin nanosheets. Similarly, the preparation of TiO_2_, ZrO_2_, Nb_2_O_5_, SnO_2_, and Ta_2_O_5_ nanostructures using alkoxide precursors in the presence of GO has also been reported.^22^ These approaches highlight the versatility of GO-assisted confined growth strategies for the fabrication of two-dimensional metal oxide nanomaterials with a controlled morphology and enhanced functional properties.

The novelty of this work lies in the development of a sensitive platform for the direct electrochemical detection of GSM. This was achieved through the synthesis of ultrathin Zn_2_Ti_3_O_8_ (ZTO) nanosheets grown on GO as a sacrificial template. Notably, this confined growth strategy yielded an unexpected crystalline phase of ZTO, which is distinct from the conventional formation of separate ZnO and TiO_2_ phases. The resulting 2D ZTO/GO architecture, combined with a strong interfacial synergy between ZTO and GO, significantly enhances the electroactive surface area and facilitates efficient charge transfer, thereby improving the overall electrochemical performance of the sensor.

The graphene oxide-templated approach serves not only as a synthetic route but also as a means to stabilize ultrathin ZTO nanosheets with superior interfacial electrochemical activity. Accordingly, a glassy carbon electrode was modified with the ZTO/GO nanocomposite and applied, for the first time, to the voltammetric detection of GSM. The electrochemical detection mechanisms were thoroughly investigated. The modified electrode exhibited high sensitivity, a low limit of detection, excellent selectivity against common interferents, and good reproducibility, underscoring its potential for the practical monitoring of geosmin in real water samples. Structural, physicochemical, and electrochemical characterizations were performed to provide detailed insights into the properties of the materials. It is worth noting that the comprehensive detail of material properties is a key point for understanding the underlying detection mechanisms and for guiding future optimizations of the ZTO-GO-based sensing platform.

## 2 Materials and Methods

### 2.1 Materials and Apparatus

Graphene oxide (GO) was produced following an adaptation of Hummers’ method.^47,48^ Sodium nitrate (NaNO_3_, 99%, Neon), sulfuric acid (H_2_SO_4_, 95–98%), potassium permanganate (KMnO_4_, 99%), and hydrogen peroxide (H_2_O_2_, 30%) were obtained from Sigma-Aldrich. Nacional de Grafite supplied micronized graphite Micrograf HC30. Hydrochloric acid (HCl, 37%, Sigma-Aldrich) was used in the purification step. Ultrapure water (resistivity 18.2 MΩ·cm, Milli-Q system, Merck Millipore) was used throughout all experiments.

The Zn_2_Ti_3_O_8_ nanosheets were synthesized as follows: 120 mg of graphite oxide was dispersed in 240 mL of absolute ethanol in a 500 mL Erlenmeyer flask and stirred at 1100 rpm for 30 min. Subsequently, 125 μL of titanium­(IV) butoxide and 78 mg of zinc acetate dihydrate (both from Sigma-Aldrich) were sequentially added. The resulting dispersion was kept under constant stirring for 20 h at room temperature (∼25 °C). The obtained material was purified by repeated centrifugation cycles, followed by washing with absolute ethanol and ultrapure water to remove unreacted precursors. The purified solid was transferred to a Falcon tube, frozen, and lyophilized. The dried product was then calcined at 480 °C for 90 min.

For comparison purposes, ZnO and TiO_2_ were synthesized using the same methodology, employing precursor concentrations of 2.96 mmol L^–1^. The metal precursors were dissolved in a GO dispersion (0.5 mg mL^–1^ in absolute ethanol), followed by overnight stirring. The resulting materials were centrifuged, washed several times with ethanol and ultrapure water, lyophilized, and calcined at 500 °C for 120 min.

The morphology and structure of the synthesized materials were characterized by scanning electron microscopy (SEM, Nova Nanolab 600, FEI Company) operated at an accelerating voltage of 5 kV, coupled with energy-dispersive X-ray spectroscopy (EDS). Transmission electron microscopy (TEM, Titan 80–300 kV, FEI Company) was performed at an accelerating voltage of 100 kV. X-ray diffraction (XRD) patterns were recorded using a diffractometer (D8 Focus diffractometer, Bruker (Germany)) with Cu Kα radiation (λ = 1.5406 Å), operating in the 2θ range of 5–80°, with a step size of 0.02°. X-ray photoelectron spectroscopy (XPS) measurements were carried out using a SPECS XPS system (base pressure of 1 × 10^–9^ mbar) equipped with a PHOIBOS 150 hemispherical electron analyzer using monochromatic Al-Ka radiation. The spectra were recorded with a pass energy of 50 and 25 eV for surveys and high-resolution measurements, respectively. The spectrometer energy scale was previously calibrated using a clean Ag single-crystal sample, setting the Ag 3d_5/2_ peak (368.2 eV), resulting in a full width at half maximum (FWHM) of 0.65 eV. An electron flood gun was employed to compensate for the electrical sample charging set to values varying from 10 to 11 eV and 40–50 μA. XPS spectra were analyzed using the CasaXPS software package,^49^ and all binding energies were calibrated by referencing the adventitious C 1s (C–C) peak to 284.8 eV. The high-resolution Zn 2p and Ti 2p regions were fitted by pseudo-Voigt Gaussian–Lorentzian product functions, GL,^30^ using a Shirley-type background.

### 2.2 GCE-Zn_2_Ti_3_O_8_ Nanosheet Preparation

The GCE (diameter: 2 mm, Metrohm) was polished with alumina slurry (1.0 and 0.3 μm), followed by thorough rinsing with deionized water. The electrode was then sonicated sequentially in 0.2 mol L^–1^ HNO_3_, ethanol, and deionized water for 5 min each to remove residual alumina.^28^ The cleanliness of the electrode surface was verified by cyclic voltammetry (CV) in 0.2 mol L^–1^ KCl solution, within a potential window from −0.2 to 1.2 V at a scan rate of 50 mV s^–1^.

The preparation of the Zn_2_Ti_3_O_8_-modified GCE involved drop-casting 20 μL of Zn_2_Ti_3_O_8_ suspension (0.3 mg mL^–1^) prepared in absolute ethanol onto the pretreated electrode surface, followed by drying at 40 °C in an oven.

### 2.3 Electrochemical Measurements

All electrochemical experiments were carried out in a conventional three-electrode cell at room temperature (∼25 °C). An Ag/AgCl electrode (3.0 mol L^–1^ KCl, Analion) was used as the reference electrode, and a platinum spiral wire (Analion) served as the counter electrode. Electrochemical measurements were performed using an AUTOLAB potentiostat/galvanostat (PGSTAT 204, Metrohm Autolab, Netherlands), controlled by NOVA 2.1.4 software. Data processing and graph plotting were conducted using NOVA 2.1.4 and Origin 2018 (OriginLab Corporation, USA), respectively.

The GSM stock solution (4 mmol L^–1^) was prepared by appropriate dilution of a certified reference material (100 μg mL^–1^, TraceCERT, Sigma-Aldrich) in ultrapure water. The supporting electrolyte was 0.1 M phosphate buffer saline (PBS) at pH 1.00.

The electrochemical behavior of GSM at the bare and Zn_2_Ti_3_O_8_, TiO_2_, and ZnO-modified GCEs was initially investigated by cyclic voltammetry (CV), aiming to evaluate the redox characteristics of the analyte and the influence of electrode surface modification on the electrochemical response. Measurements were carried out in the presence of 3.6 μg mL^–1^ GSM, within a potential window from −0.2 to 2.0 V at a scan rate of 10 mV s^–1^.

The electroanalytical performance of the proposed sensor was assessed using differential pulse voltammetry (DPV), due to its higher sensitivity and improved resolution. Analytical curves were constructed by recording the DPV response at increasing GSM concentrations under optimized conditions. DPV measurements were performed with a pulse amplitude of 50 mV, step potential of 5 mV, initial potential of +1.0 V, and final potential of +1.7 V. The linearity range, limit of detection (LOD), and quantification (LOQ) were determined from the calibration plots. The LOD was calculated based on the standard deviation and the slope of the calibration curve (LOD = 3σ/slope). The precision of the method was evaluated under intermediate-precision conditions using independently prepared Zn_2_Ti_3_O_8_-modified electrodes following the same fabrication protocol. Accuracy was assessed through recovery experiments at different spiked concentration levels.

The background current was evaluated by recording DPV curves in the blank supporting electrolyte under identical conditions and subsequently subtracting it from the analytical signal. This procedure minimizes the contribution of the oxygen evolution reaction (OER) and ensures a more accurate determination of the GSM response.

Electrochemical impedance spectroscopy (EIS) measurements were performed by using an AC amplitude of 10 mV over a frequency range from 100 kHz to 0.1 Hz, highlighting the effect of surface modification on charge transfer and diffusion processes.

## 3 Results and Discussion

### 3.1 Zn_2_Ti_3_O_8_ Nanosheet Synthesis and Characterization

Two-dimensional TiO_2_, ZnO, and Zn_2_Ti_3_O_8_ nanosheets were successfully synthesized using GO as a sacrificial template through a confined growth approach. Scheme 1 illustrates the synthetic route for the preparation of this material through a confined growth mechanism on GO. In the first step (1), Ti^4+^ and Zn^2+^ metal precursors are separately added to a GO suspension in ethanol, which is rich in oxygen-containing functional groups (−COOH, C=O, −OH, and epoxy) and exhibits a negatively charged surface (ζ-potential ≈ −50 mV), promoting the formation of the corresponding metal oxides.^49^ Electrostatic interactions and coordination between the metal ions and the functional groups of graphene oxide result in a homogeneous coating of the GO surface.^50,51^ Subsequently, the suspensions are centrifuged, washed, freeze-dried, and calcined at 500 °C during 120 min, leading to the formation of 2D TiO_2_ and ZnO nanosheets.

**Scheme 1:**
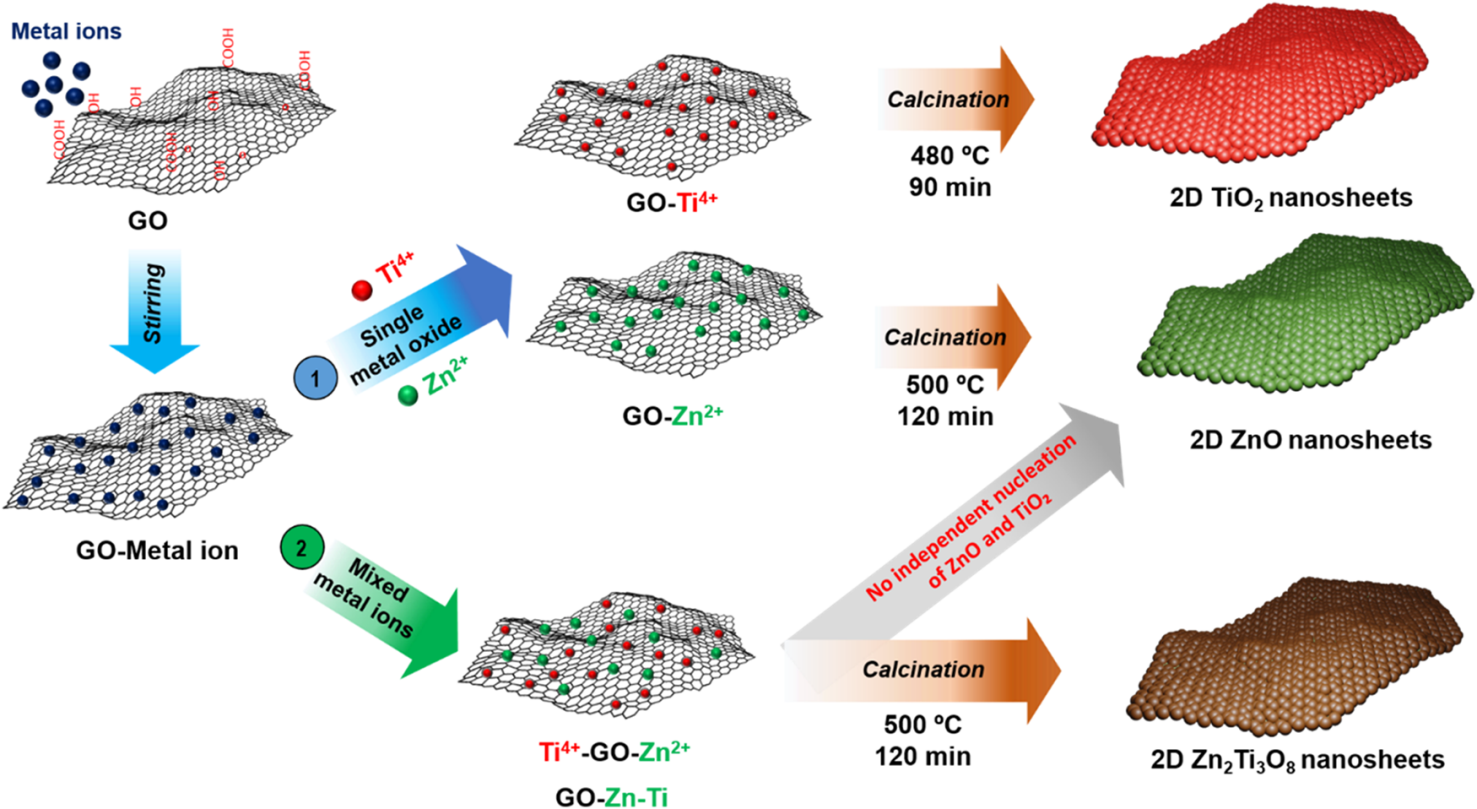
Schematic Illustration of the Synthetic Procedure for 2D TiO_2_, ZnO, and Zn_2_Ti_3_O_8_ Nanosheets

In the second step (2), the same procedure is employed, except that Ti^4+^ and Zn^2+^ precursors are simultaneously introduced into the GO suspension at 1:1 molar ratio, favoring the formation of 2D Zn_2_Ti_3_O_8_ nanosheets under calcination conditions of 480° for 90 min. As previously reported by AbdelHamid et al., the confined growth method on GO ensures that nucleation and growth of metal oxides occur exclusively at the GO–precursor interface.^52^ In the present work, when only a single cation is present in the system (step 1), either Ti^4+^ or Zn^2+^, growth on the GO surface does not alter the crystalline phase of the resulting oxides—anatase TiO_2_ (tetragonal) and hexagonal ZnO—serving primarily to control the morphology and crystallite size.^52^

In contrast, when Ti^4+^ and Zn^2+^ are introduced simultaneously, both cations are anchored within the same surface domains of GO, and their spatial proximity promotes the direct crystallization of a new mixed phase. During calcination, the forced proximity between Zn and Ti, combined with the geometric confinement imposed by GO, preferentially drives the formation of the defective spinel-phase Zn_2_Ti_3_O_8_. The cubic crystal structure of Zn_2_Ti_3_O_8_ consists of [TiO_6_] octahedra and [ZnO_4_] tetrahedra and contains cation vacancies equivalent to four Ti^4+^ ions distributed over 16 spinel lattice sites, which enhances ionic diffusion at the nanomaterial surface and, consequently, its suitability for electrochemical applications.^53^ The term “defective Zn_2_Ti_3_O_8_ nanosheets” used in this work refers to the intrinsic structural characteristics of the Zn_2_Ti_3_O_8_ phase, which is widely recognized as a defective spinel-type oxide. Unlike ideal AB_2_O_4_ spinels, Zn_2_Ti_3_O_8_ exhibits nonstoichiometry that is typically accommodated by cation vacancies and partial redistribution of Zn^2+^ and Ti^4+^ ions between tetrahedral and octahedral sites, resulting in structural disorder. This defective nature is an inherent feature of the phase itself, as reported in previous studies, and is further accentuated by the nanosheet morphology, which promotes a high density of surface and edge-related defects. Therefore, the designation “defective Zn_2_Ti_3_O_8_ nanosheets” in this study is based on the known crystallographic characteristics of Zn_2_Ti_3_O_8_ and its morphology, rather than on a direct quantitative measurement of defect density.^54^

The literature reports the synthesis of 2D ZnO/Co_3_O_4_ and ZnO/SnO_2_ nanosheets obtained via confined growth on GO using spray pyrolysis.^55^ Unlike this approach, in which metal precursors are separately anchored onto GO and subsequently combined to form heterostructures, the method proposed herein establishes a new synthetic route for producing the mixed oxide Zn_2_Ti_3_O_8_ (ZTO) with a 2D nanosheet morphology. This strategy provides enhanced control over nucleation and crystal growth, resulting in materials with well-defined architectures that are potentially promising for electrochemical applications.

The optimal calcination temperature for GO–Ti-Zn was evaluated through thermogravimetric analysis (TGA) (Figure S1d). The first-derivative TGA curve (DTG) revealed two distinct peaks at 230 and 475.8 °C. The first peak is attributed to the thermal decomposition of labile oxygen-containing functional groups on GO (hydroxyl, epoxy, carbonyl, and carbonyl groups).^56^ The second peak, more intense, corresponds to the GO combustion of the carbon skeleton. Complete removal of GO is only achieved above 600 °C. However, the objective of this work is not to eliminate the entire GO structure but rather to partially remove it while preserving the high specific surface area of the resulting material. It is worth noting that excessively high temperatures promote irreversible agglomeration of the oxide nanosheets. Thus, the study selected a calcination temperature of 480 °C for 90 min for GO-Ti-Zn and 500° for 120 min for GO-Zn and GO-Ti, to ensure complete removal of the residual precursor and excess sacrificial template while minimizing agglomeration of the nanosheets.

SEM/EDS and TEM images of the TiO_2_, ZnO, and ZTO samples are shown in Figure 1. As observed in Figure 1a–c, the TiO_2_, ZnO, and ZTO nanosheets exhibit a two-dimensional (2D) morphology similar to that of graphene oxide (GO), with smooth and homogeneous surfaces. The EDS spectra (insets in Figure 1a–c) display characteristic energy peaks corresponding to zinc (1.01 and 8.64 keV), titanium (0.45 keV), oxygen (0.52 keV), and carbon (0.25 keV), as expected. The aluminum signal arises from the sample holder. TEM images (Figure 1d–f) reveal a highly porous 2D nanosheet morphology for ZnO and ZTO (Figure 1e,f), with nanoparticle sizes ranging from 5 to 50 nm. This observation suggests that the interaction between GO functional groups and Zn^2+^ ions plays a crucial role in controlling the nucleation and spatial distribution of ZnO domains, promoting the formation of mesoporous nanosheets after calcination.^50,57^ The ZTO sample exhibits a predominantly amorphous character, as evidenced by the diffuse halo observed in the selected area electron diffraction (SAED) pattern (inset in Figure 1f), which is consistent with the XRD results (Figure 2a).

**Figure 1:**
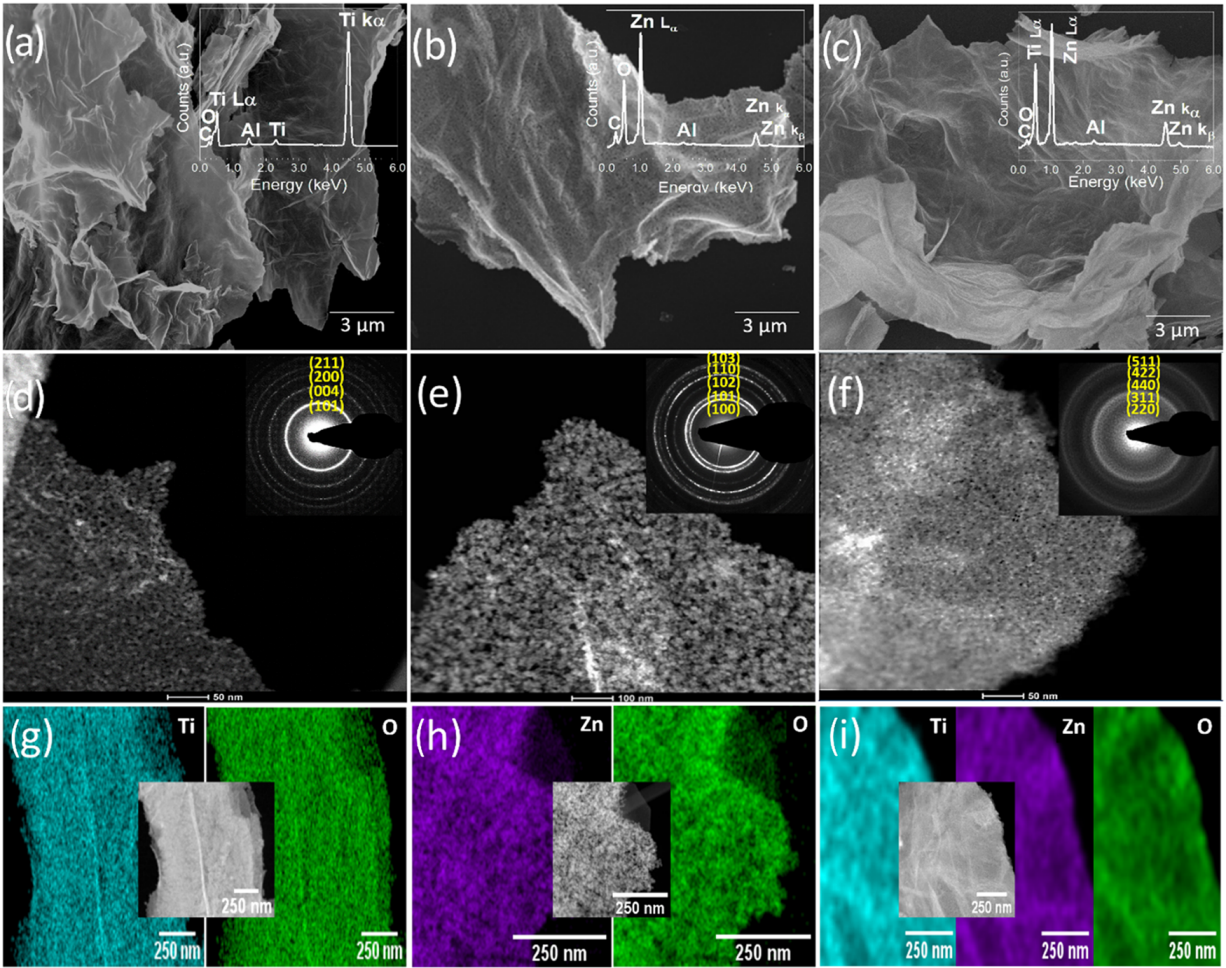
SEM images with the corresponding EDS spectra (insets) of (a) TiO_2_, (b) ZnO, and (c) Zn_2_Ti_3_O_8_, where a well-defined nanosheet-like morphology is observed; TEM images with the respective SAED patterns of (d) TiO_2_, (e) ZnO, and (f) Zn_2_Ti_3_O_8_; and EDS elemental mapping of (g) TiO_2_, (h) ZnO, and (i) Zn_2_Ti_3_O_8_.

**Figure 2:**
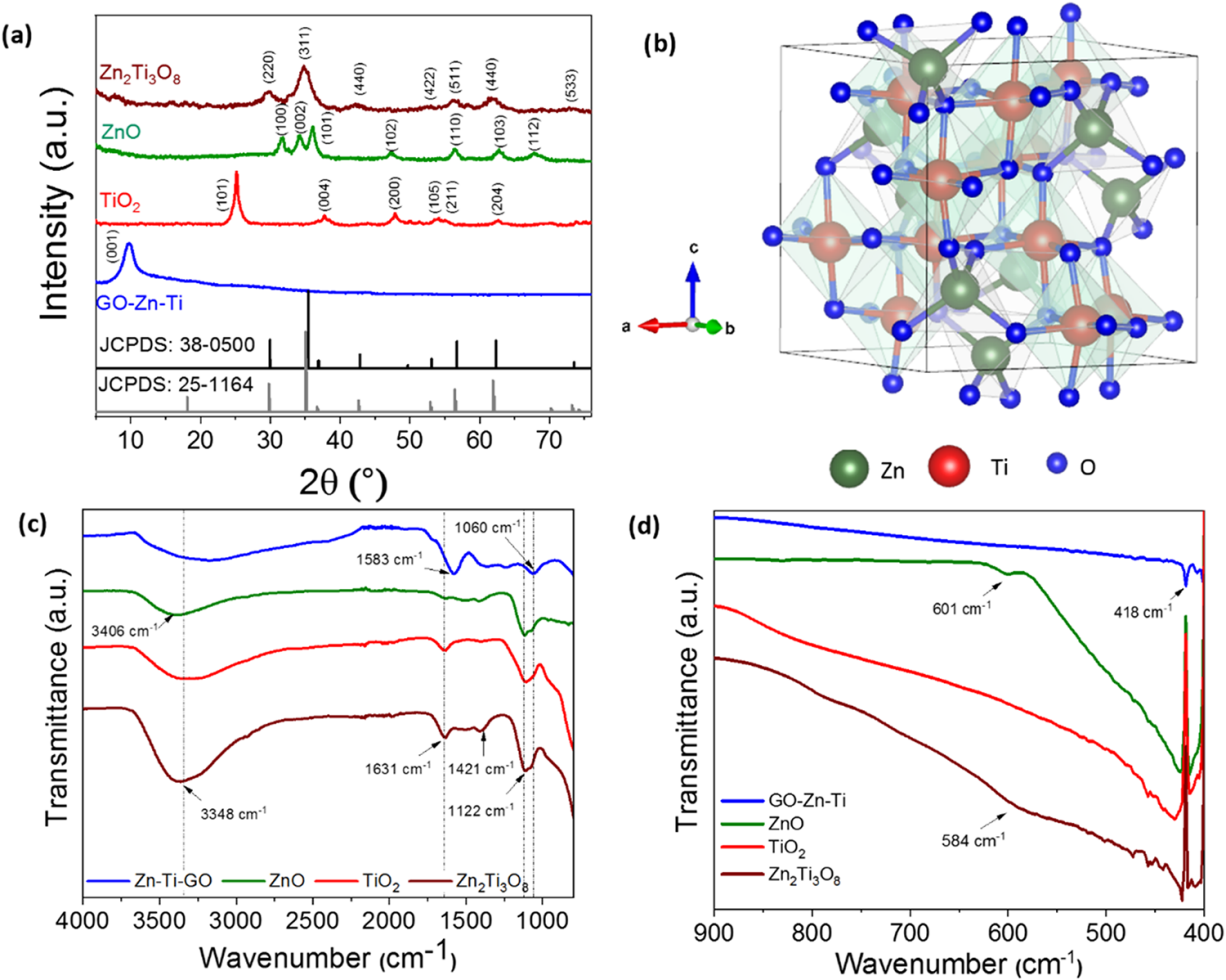
(a) X-ray diffraction (XRD) patterns of GO–Zn–Ti, ZnO, TiO_2_, and Zn_2_Ti_3_O_8_; (b) schematic representation of the crystalline structure of Zn_2_Ti_3_O_8_, generated using VESTA software;^65^ and (c, d) FTIR spectra of GO–Zn–Ti, ZnO, TiO_2_, and Zn_2_Ti_3_O_8_.

The XRD pattern of GO–Zn–Ti (Figure 2a) exhibits a single diffraction peak at 2θ = 9.76°, which is assigned to the (001) plane of GO and is associated with the interlayer spacing between graphene oxide sheets.^55^ After incorporation of the metal oxides, the diffraction patterns indicate that TiO_2_ (red line) and ZnO (green line) crystallize in the anatase and wurtzite phases, respectively, as evidenced by the characteristic diffraction peaks indexed to the (101), (004), and (200) planes of anatase TiO_2_ ^58,59^ and to the (100), (002), and (101) planes of wurtzite ZnO.^60–63^ Figure 2a it also shows the XRD pattern of the synthesized material (brown line) in comparison with the reference patterns. The diffraction peaks at 30.6, 35.8, 42.9, 53.1, 57.6, 63.2, and 73.3° are assigned to the (220), (311), (400), (422), (511), (440), and (533) crystallographic planes, respectively, corresponding to the defective cubic spinel-phase Zn_2_Ti_3_O_8_.^52^ In this structure, Zn_2_Ti_3_O_8_ is composed of octahedral [TiO_6_] and tetrahedral [ZnO_4_], possessing four missing Ti^4+^ on the 16 sites of the spinel structure.^44,53,64^ No diffraction peaks corresponding to ZnO or TiO_2_ were found, indicating phase purity.

The crystallographic features of the synthesized materials were further investigated by HRTEM (Figure S3) and correlated to the XRD results. For ZnO, the HRTEM analysis reveals lattice fringes with interplanar spacings of 0.249 and 0.303 nm, which are assigned to the (101) and (100) planes of hexagonal ZnO, respectively. These results confirm the formation of the wurtzite structure and are consistent with the XRD pattern where the characteristic reflections of ZnO are observed. The presence of multiple lattice orientations at the nanoscale indicates well-crystallized domains without a strong preferential orientation locally.

For TiO_2_, the HRTEM image shows well-defined lattice fringes with an interplanar spacing of 0.352 nm, corresponding to the (101) plane of anatase TiO_2_. This observation is in excellent agreement with the XRD results in which the (101) reflection is the most intense peak, confirming the predominance of the anatase phase in the synthesized material.

For Zn_2_Ti_3_O_8_, the HRTEM analysis reveals lattice spacings of 0.252 and 0.234 nm, both attributed to the (311) plane of the spinel-related Zn_2_Ti_3_O_8_ structure. These values are consistent with the characteristic reflections observed in the XRD pattern, which show good agreement with a spinel-type framework. The slight variation between the measured spacings suggests the presence of local lattice distortions and structural disorder, which are typical features of defective spinel systems. This behavior further supports the structural assignment of Zn_2_Ti_3_O_8_ as a defect-rich spinel-related phase, in line with the XRD analysis and comparison with the standard Zn_2_TiO_4_ structure.

Figure 2c,d present the FTIR spectra of the synthesized nanosheets. In the GO–Zn–Ti spectrum, a band at 418 cm^–1^ is attributed to the vibrational modes of metal–oxygen bonds (M = Zn, Ti), associated with the interaction between Zn^2+^ and Ti^4+^ ions and the GO matrix. The bands located at 1060 and 1583 cm^–1^ correspond to the C–O–M and C=C/C=O stretching vibrations of GO, respectively, whose variations indicate chemical interactions between the oxygen-containing functional groups and the π-conjugated system of GO and the metallic centers. The broadband centered at 3348 cm^–1^ is assigned to the O–H stretching vibrations of surface hydroxyl groups and/or adsorbed water.^66^ After calcination, the FTIR spectra of ZnO, TiO_2_, and ZTO exhibit characteristic bands of the corresponding metal oxides, with the band at 1122 cm^–1^ being associated with Zn–O–Zn, Ti–O–Ti, and Zn–O–Ti linkages.^55^ The peak at 1421 cm^–1^ is attributed to Zn–O–Zn bonds, while the bands at 601 and 584 cm^–1^ are assigned to Zn–O stretching modes and TiO_6_ octahedral stretching vibrations, respectively (Figure 2d).^67,68^ The bands observed at 1060 and 1631 cm^–1^ are related to the presence of residual C–O–M and C=C bonds resulting from the incomplete removal of GO/rGO after calcination.^68^

The surface conditions of the metal oxide nanosheets and the chemical environments of titanium, zinc, and oxygen were investigated by X-ray photoelectron spectroscopy (XPS). Figure 3a–f presents the survey and the high-resolution XPS spectra of the Ti 2p, Zn 2p, and O 1s regions. Figure 3a shows the survey spectra of the ZnO, TiO_2_, and Zn_2_Ti_3_O_8_ samples. For all samples, a small C 1s peak attributed to adventitious carbon and possibly residual rGO species is observed, together with the O 1s signal. In contrast to ZnO and TiO_2_ samples, which exhibit, as expected, either Zn or Ti core-level related peaks, respectively, for the Zn_2_Ti_3_O_8_ nanosheets, one can observe both Zn and Ti species present on the surface.

**Figure 3:**
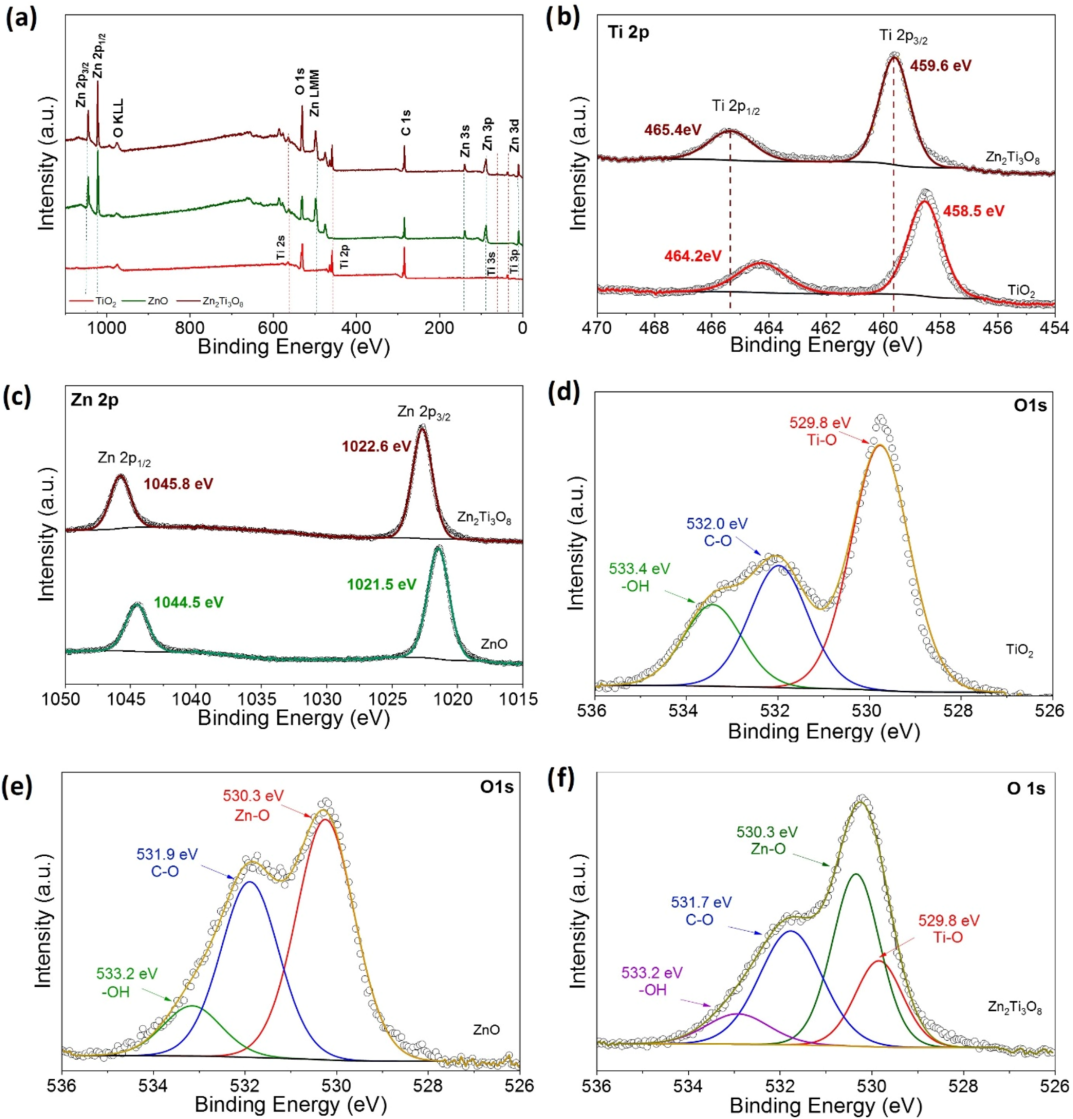
XPS spectra of TiO_2_, ZnO, and Zn_2_Ti_3_O_8_ samples: (a) survey spectra; (b) high-resolution Ti 2p spectra of TiO_2_ and Zn_2_Ti_3_O_8_; (c) high-resolution Zn 2p spectra of ZnO and Zn_2_Ti_3_O_8_; and (d–f) deconvoluted O 1s spectra of TiO_2_, ZnO, and Zn_2_Ti_3_O_8_, respectively, showing the contributions of metal–oxygen bonds, hydroxyl groups, and C–O species.

In Figure 3b, the Ti 2p_3/2_ and Ti 2p_1/2_ peaks are observed at 464.2 and 458.5 eV, respectively, with a spin–orbit splitting of 5.7 eV.^31,38,69–71^ In the Zn_2_Ti_3_O_8_ sample, the Ti 2p peaks are slightly shifted toward higher binding energies, appearing at 459.6 and 465.4 eV. This shift suggests an electronic interaction between Ti and Zn species in the mixed oxide structure, indicating changes in the local chemical environment around Ti atoms.^42^ The absence of additional peaks also confirms that titanium remains predominantly in the Ti^4+^ oxidation state in both samples. These results indicate that titanium retains the Ti^4+^ oxidation state, characteristic of octahedral coordination in TiO_6_ units, as corroborated by the O 1s spectrum (Figure 3d). Figure 3c shows the high-resolution Zn 2p spectra of ZnO and Zn_2_Ti_3_O_8_. Both materials display two peaks located at 1021.5 and 1044.5 eV for ZnO and at 1022.6 and 1045.8 eV for Zn_2_Ti_3_O_8_, which are assigned to the Zn 2p_3/2_ and Zn 2p_1/2_ states, respectively.^72,73^ The spin–orbit splitting of approximately 23 eV in both cases confirms the Zn^2+^ oxidation state.^38^ The peak associated with metal–oxygen bonds (Ti/Zn–O^2–^) is centered at approximately 530.5 eV.^53^ The high-resolution O 1s spectra reveal the presence of different oxygen chemical environments in the analyzed samples. For TiO_2_ (Figure 3d), the main peak centered at 529.8 eV is attributed to lattice oxygen bonded to titanium (Ti–O), which is characteristic of the titanium dioxide crystalline structure. The components located at higher binding energies, around 532.0 and 533.4 eV, are assigned to surface-adsorbed C–O species and hydroxyl groups (−OH), respectively, indicating the presence of surface defects and adsorbed oxygen-containing species. In the ZnO sample (Figure 3e), the dominant peak at 530.3 eV corresponds to Zn–O lattice bonds of zinc oxide. Meanwhile, the peaks observed at 531.9 and 533.2 eV are associated with surface-oxygenated species related to C–O bonds and hydroxyl groups (−OH), respectively. The presence of these components suggests the adsorption of atmospheric species and oxygen vacancies on the material surface. For the Zn_2_Ti_3_O_8_ composite (Figure 3f), the O 1s spectrum exhibits simultaneous contributions attributed to Zn–O (530.3 eV) and Ti–O (529.8 eV) bonds, confirming the formation of a mixed phase containing both metallic species. Additionally, the components centered at 531.7 and 533.2 eV are related to C–O species and surface hydroxyl groups (−OH), respectively. The relatively enhanced intensity of these surface-related components may be associated with a higher density of structural defects and oxygen vacancies generated by the interaction between Zn and Ti within the composite structure, which favors the adsorption of hydroxyl- and oxygen-containing species on the surface. These results, together with the XRD analysis, confirm that the mixed Zn_2_Ti_3_O_8_ phase was successfully synthesized.

### 3.2 Electrochemical Behavior of GCE-Zn_2_Ti_3_O_8_ Nanosheets

The electrochemical behavior of the bare GCE and the GCE-Zn_2_Ti_3_O_8_ nanosheets was evaluated by cyclic voltammetry in the absence and presence of GSM (Figure 4a), aiming to assess the electrocatalytic effect of the nanomaterial. In the blank solution, both electrodes exhibited negligible faradaic currents within the investigated potential window, indicating the absence of electroactive impurities and confirming the electrochemical stability of the modified surface. In contrast, upon addition of GSM, a well-defined anodic peak at approximately 1.5 V was observed at GCE-Zn_2_Ti_3_O_8_. No corresponding analytical signal was detected on the bare GCE under the same experimental conditions. This result demonstrates the essential role of Zn_2_Ti_3_O_8_ nanosheets in promoting the electrooxidation of GSM. The enhanced response can be attributed to the increased electroactive surface area and improved charge transfer properties provided by the nanosheet structure, which facilitate electron transfer between GSM and the electrode surface. A similar oxidation potential has been reported for GSM detection using nanocarbon electrodes decorated with Pt nanoparticles,^14^ supporting the electrochemical feasibility of the oxidation process. Overall, these results confirm the electrocatalytic activity of Zn_2_Ti_3_O_8_ nanosheets and validate their suitability for the development of a sensitive electrochemical sensor for the determination of GSM.

**Figure 4:**
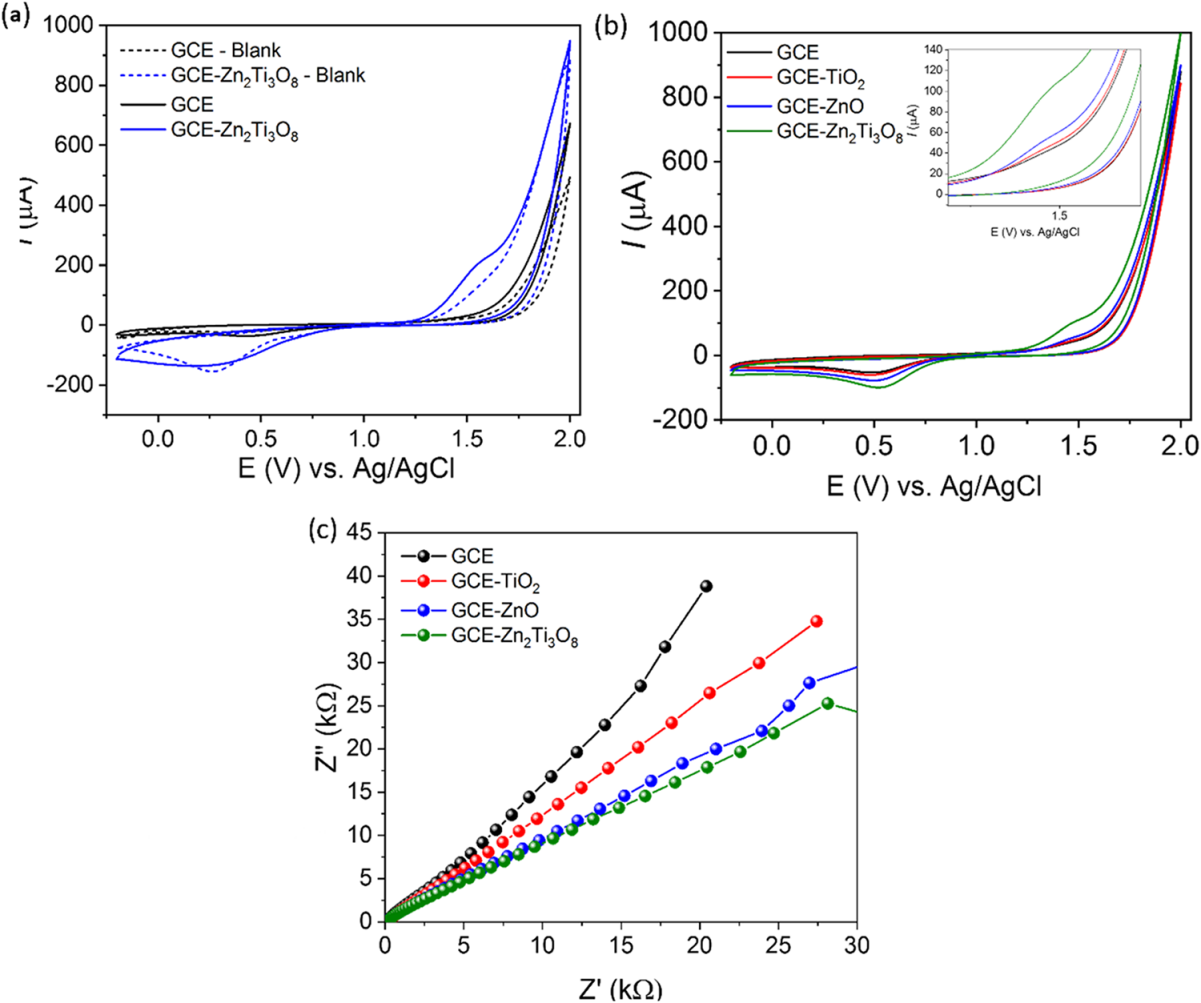
Cyclic voltammograms of (a) bare GCE and GCE-Zn_2_Ti_3_O_8_ recorded in the absence and presence of 3.6 μg mL^–1^ GSM in 0.1 M PBS (pH 1.0) at a scan rate of 100 mV s^–1^; (b) bare GCE, GCE-ZnO, GCE-TiO_2_, and GCE-Zn_2_Ti_3_O_8_ in the presence of 3.6 μg mL^–1^ GSM in 0.1 M PBS (pH 1.0) at a scan rate of 100 mV s^–1^. (c) Nyquist plots obtained from electrochemical impedance spectroscopy (EIS) measurements of the bare GCE and modified electrodes (GCE-ZnO, GCE-TiO_2_, and GCE-Zn_2_Ti_3_O_8_) recorded in a 5 mM K_3_[Fe­(CN)_6_] solution containing 0.1 M KCl as a supporting electrolyte.

The Nyquist plots (Figure 4c) exhibit a predominantly linear profile in the low-frequency region, characteristic of diffusion-controlled processes associated with Warburg-type impedance. Among the evaluated electrodes, GCE–Zn_2_Ti_3_O_8_ nanosheets present the lowest overall impedance and a reduced slope in the low-frequency region, indicating enhanced mass transport and improved interfacial charge transfer compared to the other electrodes. These results are consistent with the voltammetric findings and further corroborate the superior electrochemical performance of the Zn_2_Ti_3_O_8_-modified electrode (Figure 4b). The charge transfer resistance (*R*_ct_) could not be clearly determined due to the absence of a well-defined semicircle, indicating diffusion-controlled behavior and sluggish electron transfer kinetics.

#### 3.2.1 Optimization of Electrochemical Parameters

##### 3.2.1.1 Effect of Zn_2_Ti_3_O_8_ Loading

The amount of Zn_2_Ti_3_O_8_ deposited on the GCE surface was optimized to maximize the oxidation peak current (*I*_p_). Different loadings (1.5, 3.0, 6.0, and 9.0 μg) were evaluated by cyclic voltammetry (Figure 5a). The peak current increased progressively with increasing Zn_2_Ti_3_O_8_ loading, reaching a maximum at 6.0 μg. This enhancement can be attributed to the increased density of electroactive sites and improved surface roughness provided by the nanosheet structures, which favor electron transfer and analyte interaction. However, a further increase to 9.0 μg resulted in a decrease in the peak current. This behavior is likely associated with the formation of a thicker nanomaterial layer, which may hinder charge transfer kinetics and partially block the conductive glassy carbon surface. Excessive loading can also reduce mass transport efficiency due to increased film compactness.

**Figure 5:**
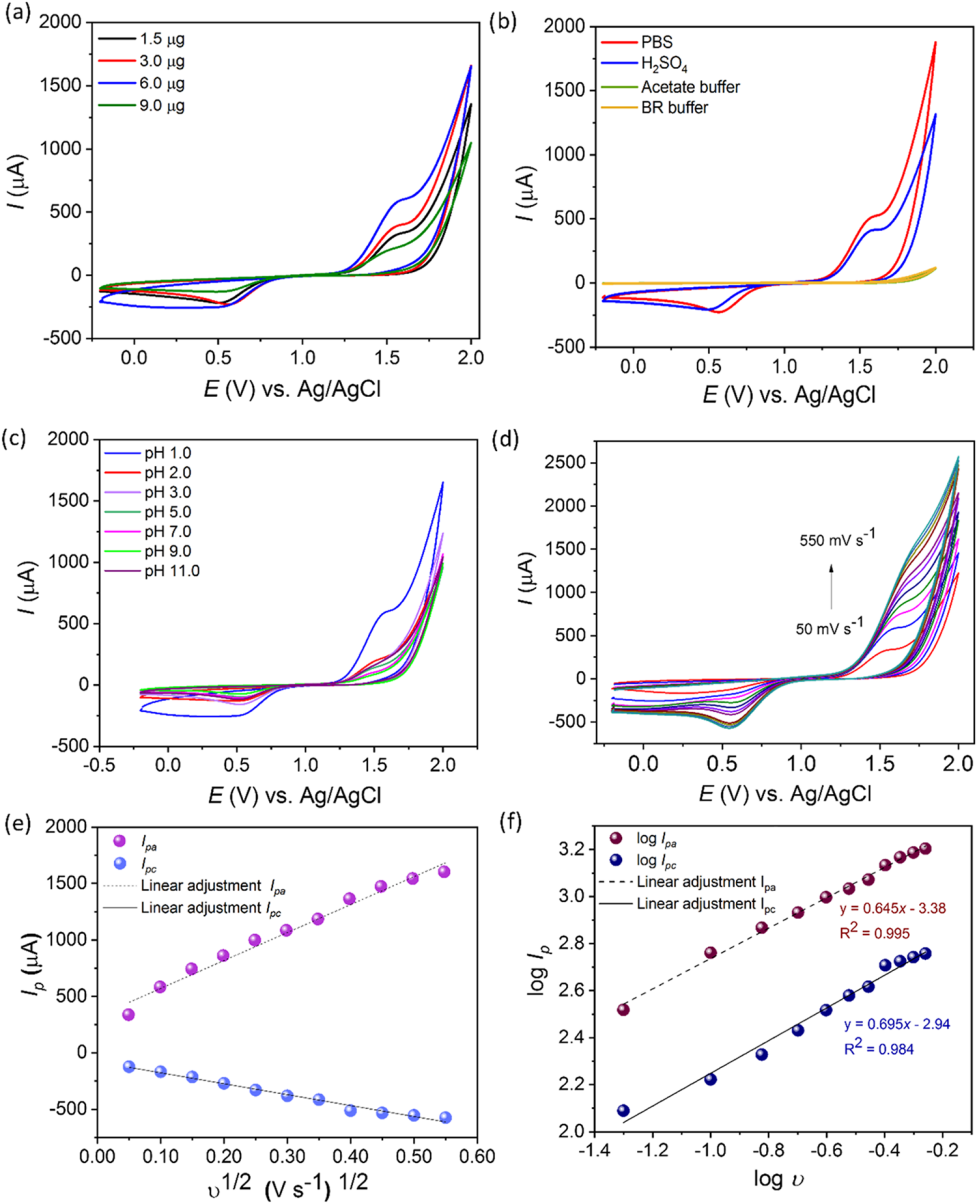
Cyclic voltammograms of the GCE modified with Zn_2_Ti_3_O_8_ nanosheets in the presence of 0.02 μmol L^–1^ of GSM under different conditions: (a) nanosheet loading, (b) supporting electrolyte, (c) pH, and (d) scan rate. (e) Linear relationship between the square root of the scan rate (ν^1/2^) and the anodic and cathodic peak currents. (f) Log–log plots of peak current (*I*_p_) versus scan rate (ν). Experimental conditions: CV measurements were carried out using 3.6 μg mL^–1^ GSM in 0.1 M PBS (pH 1.0) within a potential window from 0.20 to 2.0 V. Experiments in (a–c) were performed at a scan rate of 100 mV s^–1^, while in (d), scan rates ranged from 0.05 to 0.55 V s^–1^.

These results indicate the existence of an optimal loading that balances catalytic activity and electrical conductivity, suggesting a synergistic interaction between the glassy carbon substrate and the Zn_2_Ti_3_O_8_ nanosheets in the electrochemical oxidation of GSM. Therefore, 6.0 μg was selected as the optimal modification amount for subsequent experiments.

##### 3.2.1.2 Influence of the Supporting Electrolyte

The nature of the supporting electrolyte plays a crucial role in electrochemical processes, as it can influence ionic conductivity, interfacial charge transfer, and the formation of reactive species. Aiming at enhancing the electrochemical performance in the oxidation of organic contaminants, solutions containing sulfate, nitrate, perchlorate, and chloride are often used as supporting electrolytes.^74–78^ In this study, different electrolytes were evaluated to optimize the oxidation signal of GSM, namely phosphate buffer saline (PBS), Britton–Robinson buffer (BR buffer), 0.1 M acetate buffer, and 0.01 M H_2_SO_4_ (Figure 5b). The results clearly demonstrate that the electrolyte composition significantly affects the electrochemical response. The highest peak currents were obtained in H_2_SO_4_ and PBS, whereas no well-defined oxidation signal was observed in acetate or BR buffers. This behavior indicates that both the ionic environment and the electrolyte constituents strongly influence the oxidation process.

The superior performance observed in H_2_SO_4_ may be attributed to the highly acidic medium, which can facilitate proton-coupled electron transfer processes and enhance the oxidation kinetics of GSM.^45^ Additionally, sulfate-containing media have been reported to promote the formation of reactive species under electrochemical conditions, potentially contributing to an enhanced oxidation efficiency.^74^

In the case of PBS, the improved response may be associated with the presence of chloride ions (NaCl in its composition). Chloride ions can participate in electrochemical reactions, leading to the formation of active chlorine species (e.g., HOCl), which are strong oxidants and may contribute to signal enhancement. Similar effects have been reported in studies involving electrochemical oxidation of organic contaminants, where chloride-containing electrolytes improved degradation efficiency.^75,76,78^

Conversely, the absence of a significant signal in acetate and BR buffers suggests that these media do not favor the oxidation pathway of GSM under the investigated conditions, possibly because of lower conductivity, buffering interactions, or less favorable interfacial kinetics.

On the basis of these results, PBS was selected as a suitable supporting electrolyte for subsequent studies.

##### 3.2.1.3 Effect of pH

The influence of solution pH on the electrochemical detection of GSM was systematically investigated in PBS over a pH range from 1.0 to 11.0 (Figure 5c). The results show a strong dependence of the oxidation response on the acidity of the medium. As the pH decreased, a significant increase in the oxidation peak current was observed with the highest response obtained at pH 1.0. Conversely, under neutral and alkaline conditions, the peak current progressively decreased. This behavior indicates that acidic medium favors the electrochemical oxidation of GSM. The enhanced response at low pH suggests the involvement of protons in the oxidation mechanism, indicating a possible proton-coupled electron transfer process. In strongly acidic environments (pH < 5.0), changes in the molecular stability and protonation state of GSM may increase its reactivity toward oxidation, thereby facilitating electron transfer at the electrode interface.^79–82^ Additionally, a higher proton availability can improve charge transfer kinetics and reduce activation barriers. In contrast, under alkaline conditions, the reduced availability of protons and possible changes in the GSM speciation may hinder the oxidation process, leading to lower peak currents. On the basis of these findings, pH 1.0 was selected as the optimal condition for subsequent electrochemical measurements.

##### 3.2.1.4 Scan Rate Study and Kinetic Evaluation

The effect of scan rate (ν) on the electrochemical behavior of GSM was investigated by cyclic voltammetry over the range of 0.05–0.55 V s^–1^ (Figure 5d). Both anodic and cathodic peaks are observed, indicating the occurrence of a redox process at the modified electrode surface.

For a reversible system, several criteria are typically satisfied: (i) the peak current (*I*_p_) is proportional to the square root of the scan rate (*I*_p_ ∝ ν^1/2^); (ii) the ratio between anodic and cathodic peak currents (*I*_pa_/*I*_pc_) is close to unity and independent of ν; and (iii) the peak-to-peak separation (Δ*E*_p_ = *E*_pa_ – *E*_pc_) remains constant and approaches the theoretical value of 59 mV/n at 25 °C, where n is the number of electrons involved.^83,84^ As shown in Figure 5e, both anodic and cathodic peak currents increase linearly with ν^1/2^, with correlation coefficients (*R* ^2^) of 0.982 and 0.986, respectively. This behavior indicates that mass transport plays an important role in the electrochemical process.

However, the reversibility criteria are not fully satisfied. The peak-to-peak separation (Δ*E*_p_) increases from 0.99 to 1.18 V as the scan rate increases (Table S2), which is significantly higher than the theoretical value expected for a reversible system. Moreover, the *I*_pa_/*I*_pc_ ratio ranges from 2.66 to 3.46 and remains markedly different from unity over the entire scan rate range. These findings indicate that the redox process of GSM at the modified electrode is not electrochemically reversible but rather quasi-irreversible. Additionally, both anodic and cathodic peak potentials shift with increasing scan rate, further supporting the kinetic limitation of the electron transfer process.

To elucidate the charge transfer mechanism, the relationship between log *I*_p_ and log ν was evaluated (Figure 5f). In such analysis, a slope of 0.5 indicates a diffusion-controlled process, whereas a slope of 1.0 suggests adsorption control.^84^ The slopes obtained for the anodic and cathodic processes were 0.645 and 0.695, respectively. These values indicate that the electrochemical process is governed by a mixed control mechanism, involving both diffusion and adsorption contributions. The number of electrons involved in the oxidation of GSM was estimated using the Laviron equation from the slope of the *E*_p_ vs log­(ν) plot. The calculated value suggests that approximately one electron is involved in the rate-determining step of the oxidation process. The surface coverage (Γ) was estimated as 3.8 × 10^–8^ mol cm^–2^, assuming an adsorption-controlled process (*R* = 0.96). It should be noted that the calculation was based on the geometric electrode area, and therefore, the obtained value may be underestimated.

Overall, the scan rate study demonstrates that GSM oxidation at the Zn_2_Ti_3_O_8_-modified GCE follows a quasi-irreversible electron transfer process under mixed diffusion–adsorption control.

#### 3.2.2 Morphological and Elemental Characterization of GCE-Zn_2_Ti_3_O_8_

The surface morphology of the bare and modified electrodes was investigated by scanning electron microscopy (SEM), and their elemental compositions were analyzed by energy-dispersive X-ray spectroscopy (EDS) (Figure 6).

**Figure 6:**
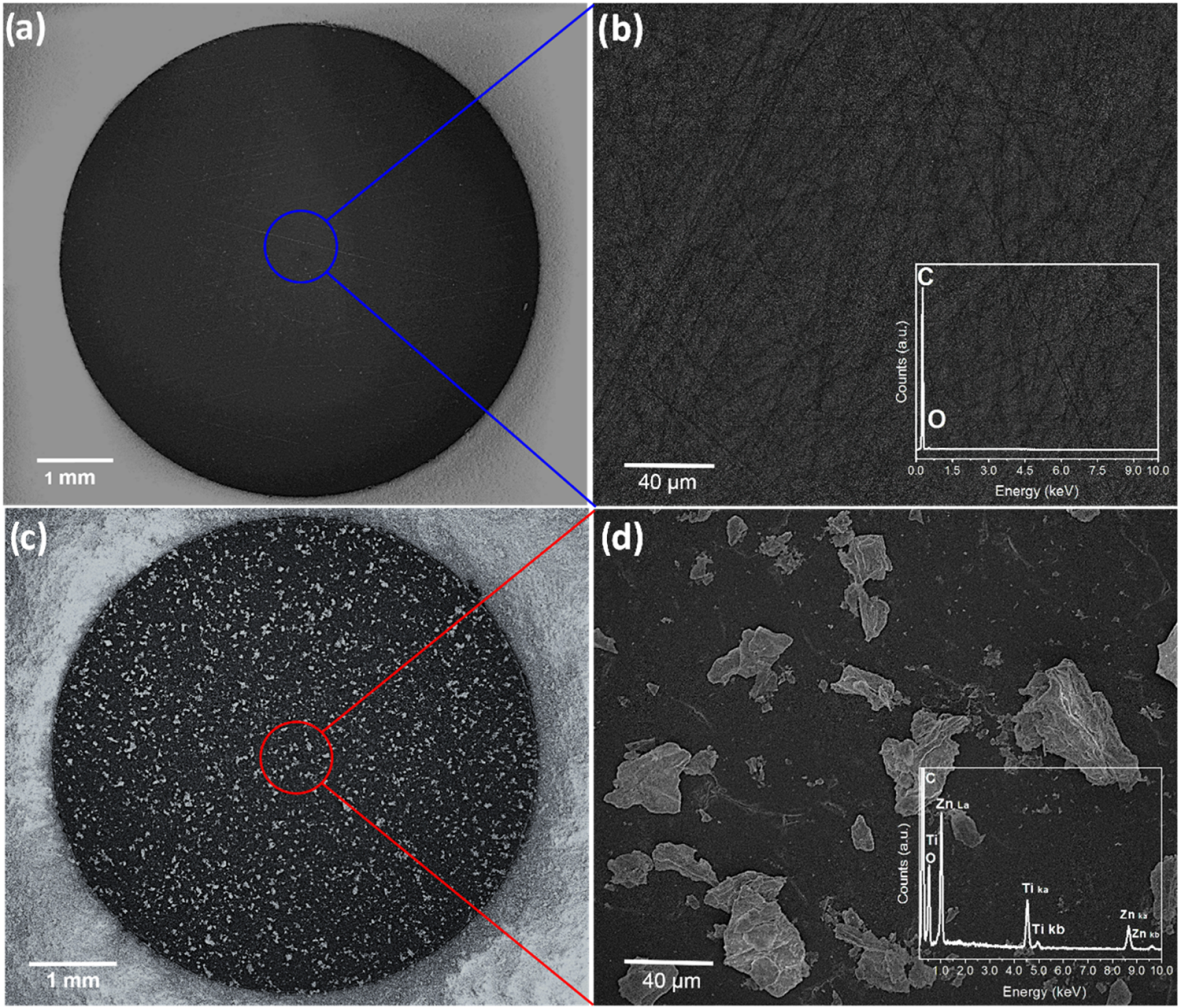
SEM images of (a) bare GCE and (b) its higher magnification view with the corresponding elemental composition shown in the inset; (c) GCE modified with Zn_2_Ti_3_O_8_ nanosheets, and (d) its higher magnification view with the corresponding elemental composition shown in the inset.

The bare GCE exhibits a relatively smooth and homogeneous surface with shallow polishing grooves, characteristic of mechanically polished glassy carbon (Figure 6a,b).^30^ The corresponding EDS spectrum (inset, Figure 6b) shows only the presence of carbon and oxygen, confirming the expected composition and the absence of contaminants on the electrode surface.

After modification with Zn_2_Ti_3_O_8_ nanosheets, significant morphological changes are observed (Figure 6c,d). The modified electrode surface becomes rougher and is partially covered by sheet-like structures, consistent with the nanosheet morphology of the synthesized material. These structures increase the surface heterogeneity and are expected to enhance the electroactive area of the electrode. The EDS spectrum of the modified surface (inset, Figure 6d) confirms the presence of Zn, Ti, and O signals in addition to carbon from the substrate, demonstrating the successful immobilization of Zn_2_Ti_3_O_8_ on the GCE surface. The simultaneous detection of these elements, without extraneous impurities, indicates that the modification process was effective and that the nanomaterial was properly anchored onto the electrode interface.

The observed morphological and compositional changes corroborate the formation of GCE-Zn_2_Ti_3_O_8_. The thickness and morphology of the Zn_2_Ti_3_O_8_ nanosheets play crucial roles in determining the electrochemical performance of the modified electrode. Thinner nanosheets provide a significantly higher surface-to-volume ratio, which increases the number of exposed active sites available for the GSM adsorption and oxidation. This enhanced surface accessibility facilitates a stronger interaction between the analyte and the electrode, contributing to higher sensitivity. Moreover, the two-dimensional nanosheet morphology promotes efficient charge transport by reducing the electron and ion diffusion distances within the material. This structural feature minimizes resistance and accelerates the electron transfer kinetics at the electrode/electrolyte interface. In addition, nanosheets with reduced thickness are more likely to exhibit a higher density of structural defects, such as cation vacancies and disordered regions, which can serve as additional active sites and further improve the catalytic activity. The combined effects of high surface area, abundant active sites, and improved charge transport result in a more efficient electrochemical response. Therefore, the observed enhancement in the sensing performance can be directly correlated with the optimized nanosheet thickness and morphology, highlighting the importance of structural control in the design of high-performance electrochemical sensors.

### 3.3 Analytical Performance: Linear Range and LOD

Figure 7a presents the differential pulse voltammetric responses of the GCE/Zn_2_Ti_3_O_8_ electrode toward increasing concentrations of GSM (0.36–3.6 μg mL^–1^) in 0.01 mol L^–1^ PBS (pH 1.0). A progressive increase in the anodic peak current is observed with increasing analyte concentration, indicating a concentration-dependent electrochemical oxidation process without noticeable peak distortion or potential shift.

**Figure 7:**
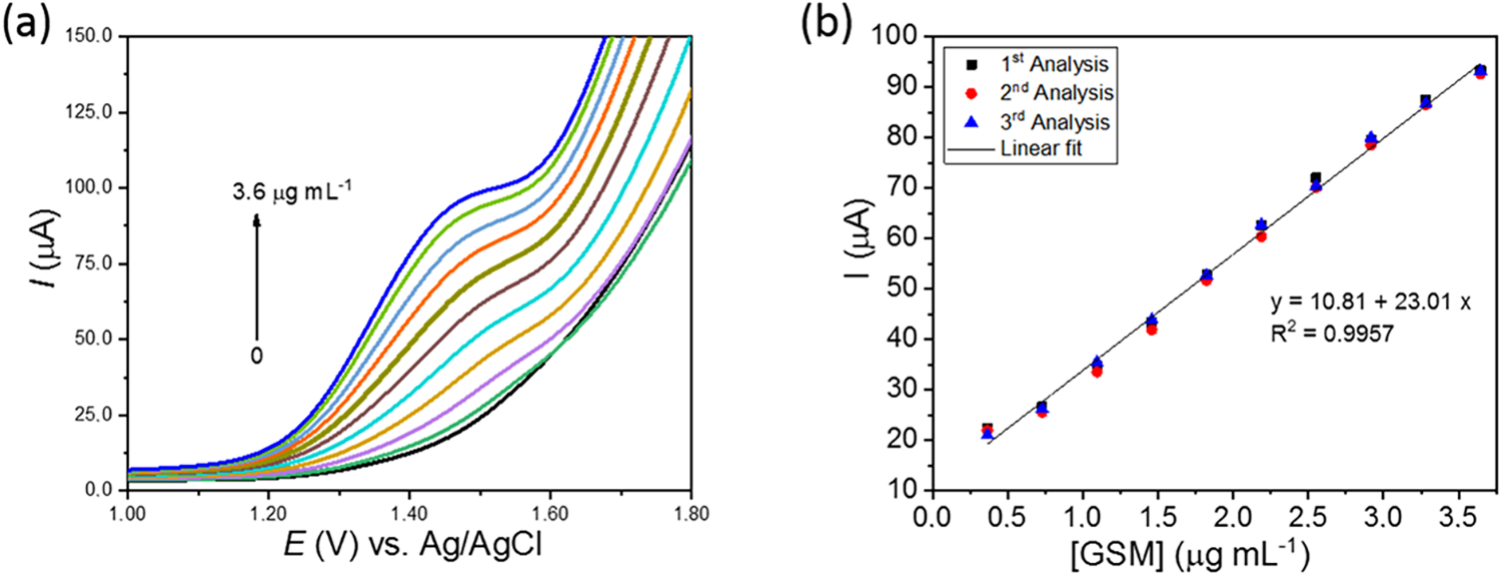
(a) Analytical curve obtained for the GCE modified with Zn_2_Ti_3_O_8_ nanosheets toward GSM in the concentration range from 0 to 3.6 μg mL^–1^ and (b) corresponding linear fit. Experimental conditions: differential pulse voltammetry (DPV) measurements were carried out in 0.1 M PBS (pH 1.0) using a potential window from +1.0 to +1.7 V, with a pulse amplitude of 50 mV, pulse width of 0.025 V, and step potential of 5 mV.

The analytical curve (Figure 7b) was constructed by plotting the peak current versus GSM concentration. All measurements were performed in triplicate. The data were statistically evaluated using Grubbs’ test for outlier detection, and no anomalous values were identified. Homogeneity of variances was verified using Cochran’s test at the 95% confidence level (*G*_calculated_ = 0.247 < *G*_critical_ = 0.445, *k* = 10, *n* = 3), confirming homoscedastic behavior across the studied concentration range. Linear regression analysis yielded the equation *I* (μA) = 10.81 + 23.01 [GSM], with a determination coefficient (*R* ^2^) of 0.9957, demonstrating excellent linear correlation. The slope value reflects the high sensitivity of the Zn_2_Ti_3_O_8_-modified electrode toward GSM oxidation.

The limits of detection (LOD) and quantification (LOQ) were calculated based on the statistical criteria, using the standard deviation of regression (0.96 μA) and the slope (23.01 μA mL μg^–1^). The calculated LOD and LOQ were 0.09 and 0.28 μg mL^–1^, respectively. These values are consistent with the visually estimated limits (0.08 and 0.27 μg mL^–1^), confirming the reliability of the statistical approach.

Although only a limited number of electrochemical studies have addressed GSM detection, most reported strategies rely on indirect sensing mechanisms or hybrid analytical systems. For example, Li et al. employed a molecularly imprinted membrane-modified GCE, in which GSM binding induces signal attenuation rather than direct electrochemical oxidation, achieving detection limits in the ng L^–1^ range. Likewise, a Pt–C film electrode coupled to an HPLC-ECD system provided enhanced sensitivity but still required chromatographic separation. In contrast, the present work demonstrates the direct electrochemical oxidation of GSM at a nanostructured Zn_2_Ti_3_O_8_-modified electrode without any preconcentration or separation step, offering a simple and fully electrochemical alternative.

It is acknowledged that the obtained detection limit (0.09 μg mL^–1^) remains higher than the odor threshold of GSM in natural waters, typically reported in the ng L^–1^ range. Nevertheless, unlike previously reported ultrasensitive approaches based on molecular imprinting, signal suppression, or chromatographic coupling, the proposed strategy relies solely on the intrinsic electrocatalytic activity of the Zn_2_Ti_3_O_8_ nanosheets toward direct analyte oxidation. Thus, this study establishes a proof-of-concept platform for straightforward electrochemical sensing, combining good linearity, reproducibility, and operational simplicity and providing a solid basis for future sensitivity enhancement through surface engineering or other strategies.

### 3.4 Precision and Accuracy Assessment

The precision of the method was assessed under intermediate-precision conditions using independently prepared modified electrodes following the same fabrication protocol. As shown in Table S3, the relative standard deviation (RSD) values ranged from 0.4 to 2.9% across the evaluated concentration levels. These low RSD values indicate excellent repeatability and confirm the high reproducibility of the electrode preparation and the measurement procedure. The absence of systematic variation across the concentration range further suggests homoscedastic behavior and stable analytical performance.

Accuracy was evaluated through recovery experiments at different spiked concentration levels. The recovery values ranged from 95 to 119%. For most concentration levels (≥0.7 μg mL^–1^), recoveries were within the typically accepted range of 95–105%, demonstrating satisfactory trueness of the method. The slightly higher recovery observed at the lowest concentration level (0.4 μg mL^–1^) may be attributed to increased relative uncertainty near the lower quantification limit. Overall, the results confirm that the proposed voltammetric method provides reliable quantification of GSM with acceptable accuracy and precision according to commonly adopted validation criteria.

### 3.5 Selectivity and Interference Study

The interference analysis was evaluated by investigating the current signal from the oxidation of interfering agents in the studied potential range. Thus, the interference of contaminants commonly found in aqueous matrices, 2-methylisoborneol (2-MIB), urea, sodium nitrate, and ferric chloride at environmentally relevant concentrations, was evaluated. The results (Figure 8) demonstrate that the anodic response of the GCE-Zn_2_Ti_3_O_8_ electrode remains statistically unchanged in the presence of common inorganic and organic species. The current variation remains within the experimental standard deviation, indicating negligible competitive oxidation or surface-blocking effects. This behavior suggests that the Zn_2_Ti_3_O_8_ nanosheet interface promotes selective electrochemical oxidation of GSM, likely due to favorable surface interactions and the absence of overlapping redox processes within the applied potential window.

**Figure 8:**
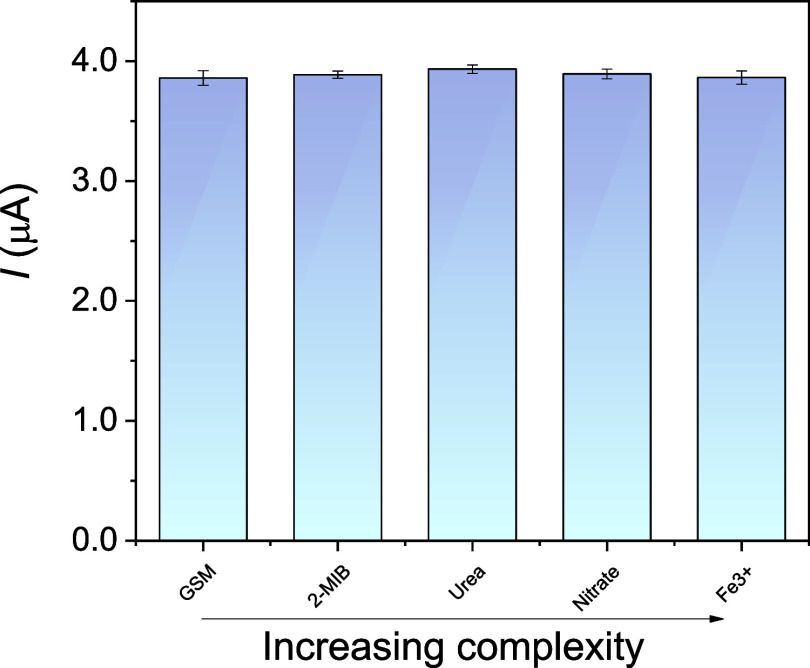
Results of the interference study for the GCE modified with Zn_2_Ti_3_O_8_ nanosheets toward GSM, evaluating the effect of potential interfering species on the analytical signal. Experimental conditions: Measurements were performed by DPV in 0.1 M PBS (pH 1.0) containing 0.02 μmol L^–1^ GSM in the absence and presence of interfering compounds at a concentration ratio of 1:10 (GSM:interferent). The analytical response was based on peak currents (*I*_p_), obtained in triplicate, and the relative signal variation (%) was used to assess selectivity.

### 3.6 Proposed Mechanism for the GSM Oxidation

The electrochemical oxidation of GSM at the Zn_2_Ti_3_O_8_-modified electrode can be described as an adsorption-controlled process, followed by electron transfer. Initially, GSM molecules are adsorbed onto the surface of the Zn_2_Ti_3_O_8_ nanosheets, which provides a high surface area and abundant active sites (Scheme 2). This adsorption is facilitated by the interaction of the hydroxyl group of the GSM with the oxide surface. The enhanced electrochemical performance can be attributed to the defective nature of Zn_2_Ti_3_O_8_, which is characterized by cation vacancies, oxygen defects, and structural disorder. These features increase the density of electronic states and create active catalytic sites that facilitate charge transfer at the electrode/electrolyte interface. As a result, the activation energy for electron transfer is reduced, leading to a decrease in the overpotential. Additionally, the nanosheet morphology promotes efficient mass transport and short diffusion pathways, further contributing to improved sensing performance. Although a complete mechanistic elucidation would require density functional theory (DFT) calculations, a plausible oxidation pathway for GSM can be proposed on the basis of its molecular structure and the surface properties of defective metal oxides. Upon adsorption, the oxidation likely proceeds through the formation of a surface-bound alkoxy intermediate, followed by proton-coupled electron transfer (PCET), generating oxidized species, such as ketones or fragmented intermediates. The presence of oxygen vacancies and mixed-metal cations (Zn^2+^/Ti^4+^) enhances adsorption strength and stabilizes intermediate species, further facilitating the oxidation process.

**Scheme 2:**
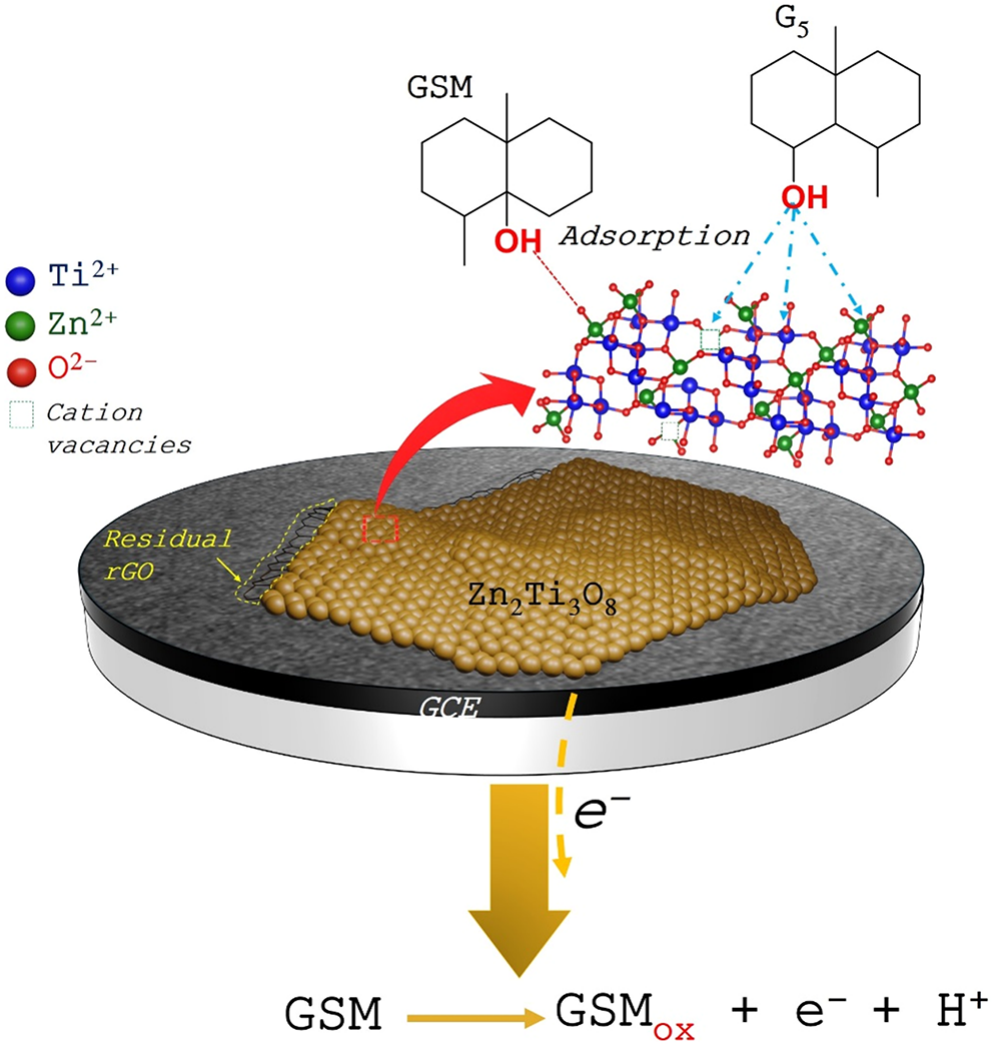
Schematic Representation for the Oxidation Route of GSM at GCE-Zn_2_Ti_3_O_8_

The superior electrochemical performance of Zn_2_Ti_3_O_8_, compared to those of ZnO and TiO_2_, is attributed to the synergistic interaction between Zn^2+^ and Ti^4+^ sites, which increases the density of active sites and promotes the adsorption of GSM and G5 via hydrogen bonding and coordination. Mixed oxides also exhibit modified electronic structures and enhanced charge transfer, while residual rGO increases the conductivity and facilitates electron transfer during oxidation.^54^

Additionally, cationic vacancies act as active centers, promoting charge mobility and adsorption.^85–87^ Zn_2_Ti_3_O_8_ also exhibits high structural stability under acidic conditions, maintaining its crystallinity under electrochemical stress.^65^ Together, these factors enhance electrocatalytic activity and effective surface area, resulting in higher sensitivity and lower detection limits.^65^

According to the literature^74,88^ and based on the observations obtained from cyclic voltammetry analyses, a mechanism for the oxidation process was proposed (Figure 9). The electrochemical oxidation of GSM may proceed through two distinct pathways (1 and 2). In the first pathway, the reaction begins with the direct oxidation of the alcohol, leading to the loss of a methylene fragment and formation of intermediate G1. The formation of G2 was owing to α-hydrogen abstraction and β-scission on GSM. G2 was further oxidized to G6 via an elimination reaction.^74^ The increased conjugation achieved at this stage facilitates further oxidative transformations that promote the progressive cleavage of the bicyclic system, ultimately generating small molecules, predominantly CO_2_ and H_2_O.^89^ In pathway 2, the strongly acidic medium first promotes protonation of the hydroxyl group in GSM, enabling an E2-type elimination that avoids the formation of an unstable bridgehead carbocation, a well-known limitation in rigid bicyclic terpenoids.^90^ The resulting alkene (G4), which is highly strained, undergoes rapid rehydration to produce G5, a more stable species capable of undergoing anodic oxidation. From this point, electrochemical activation promotes successive oxidation and radical processes that facilitate β-cleavages within the carbocyclic framework, steps favored by strain relief and the formation of partially conjugation-stabilized intermediates. These events yield the carbonyl species G6–G8, which exhibit reduced structural complexity and increased susceptibility to further oxidation, progressing toward smaller fragments and ultimately CO_2_ and H_2_O.^74^ The resulting alkene (G4), which is highly strained, undergoes rapid rehydration to yield G5, a more stable species capable of undergoing anodic oxidation. From this stage, electrochemical activation promotes successive oxidation and radical processes that facilitate β-cleavages within the carbocyclic framework, events favored by strain relief and the formation of partially conjugation-stabilized intermediates. These transformations generate the carbonyl species G6–G8, with G8 being formed with the destruction of the hexatomic ring. Under continued electro-oxidative conditions, these intermediates are subsequently mineralized into CO_2_ and H_2_O.

**Figure 9:**
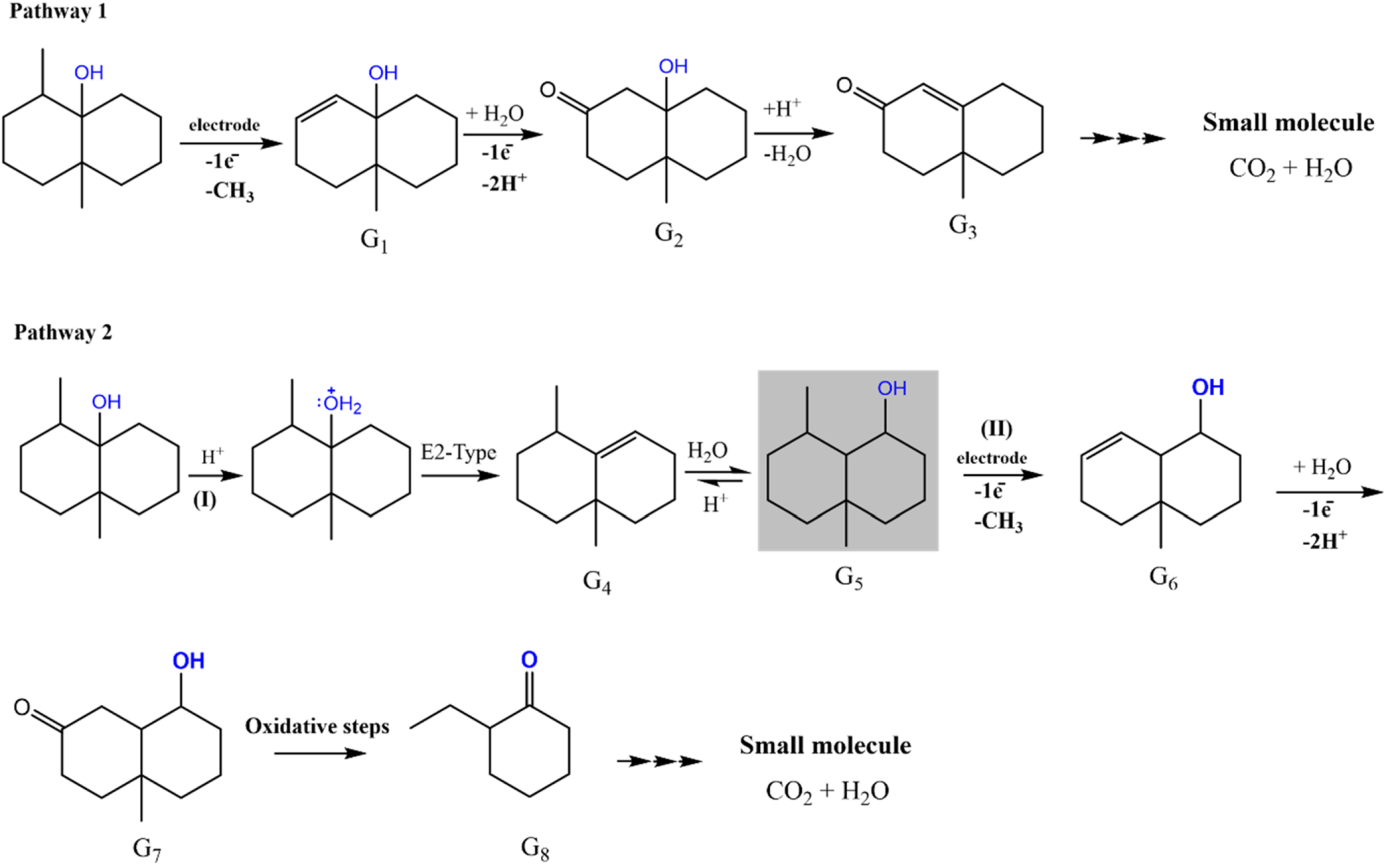
Proposed electrochemical oxidation mechanism of GSM at the GCE modified with Zn_2_Ti_3_O_8_ nanosheets, highlighting pathway 1 (inactive under the investigated conditions) and pathway 2 (active at pH ≤ 2).

## 4 Conclusions

This study establishes Zn_2_Ti_3_O_8_ nanosheets as an effective metal oxide platform for the direct electrochemical oxidation of GSM, advancing the design of functional interfaces for environmental sensing. The graphene oxide-templated synthesis enabled the formation of a stable, high-surface area architecture that promoted charge transfer and facilitated the anodic oxidation process, yielding a linear and reproducible analytical response with satisfactory interference tolerance.

Importantly, this work demonstrates that direct GSM oxidation can be achieved using a single-component oxide material, without the need for noble metal decoration or complex hybrid nanocomposites. Although the detection limits (0.08 and 0.27 μg mL^–1^) are higher than those reported for some indirect strategies, the structural simplicity, low-cost composition, and robust fabrication route position the Zn_2_Ti_3_O_8_-modified electrode as a scalable and practically viable sensing platform. Beyond geosmin detection, the findings highlight the broader potential of engineered ternary oxide nanosheets as tunable electrocatalytic interfaces. This work therefore contributes to the rational development of metal oxide-based electrochemical systems for environmental monitoring and expands the application scope of Zn_2_Ti_3_O_8_ nanostructures in interfacial electrochemistry.

## Supplementary Material



## Notes

TGA analysis of GO-based materials (GO, GO–Ti, GO–Zn, and GO–Zn–Ti); morphological and structural characterization of GO by SEM/EDS, XRD, Raman, XPS (survey and high-resolution C 1s), and FTIR; HRTEM images with lattice fringe analysis and interplanar spacing determination for TiO_2_, ZnO, and Zn_2_Ti_3_O_8_; electrochemical parameters derived from cyclic voltammetry at different scan rates for GSM at GCE–Zn_2_Ti_3_O_8_; and recovery and precision results of the voltammetric method (PDF)
