# Supplementary material for: Direct Electrochemical Detection of Geosmin Using Zn2Ti3O8 Nanosheets Grown on Graphene Oxide
Source: ACS Omega. 2026 Jun 10;11(24):35700–17. doi: 10.1021/acsomega.6c02052 (PMC13294887; doi:10.1021/acsomega.6c02052)
Supplement: Supplementary file 1 [file ao6c02052_si_001.pdf]

Supporting Information

Direct Electrochemical Detection of Geosmin Using $Zn_2Ti_3O_8$ Nanosheets Grown on Graphene Oxide

Nádia Cristina da Silva Iack [1], Kelly Leite dos Santos Castro Assis [2,*], Warley Cirqueira Machado [2], Druval Santos de Sá [2], Thayane Almeida de Medeiros [2], Carolina Carvalho de Mello [2], Maybi Falker Sampaio [2], Maria Luiza de Araujo Dorneles [3], Fernando Loureiro Stavale Junior [3], Adriana Maria da Silva [2], Braulio Soares Archanjo [1,2] and Carlos Alberto Achete [1,2]

[1] Post-Graduate Metrology and Technology Program, National Institute of Metrology, Quality and Technology, Duque de Caxias, RJ, 25250-020, Brazil.
[2] Material's Division, National Institute of Metrology, Quality and Technology, Duque de Caxias, RJ, 25250-020, Brazil.
[3] Brazilian Center for Research in Physics, Rio de Janeiro, RJ, 22290-180, Brazil
*Correspondence: klcastro@colaborador.inmetro.gov.br

Figure S1. TGA analysis of (a) GO, (b) GO-Ti, (c) GO-Zn and (d) GO-Zn-Ti.

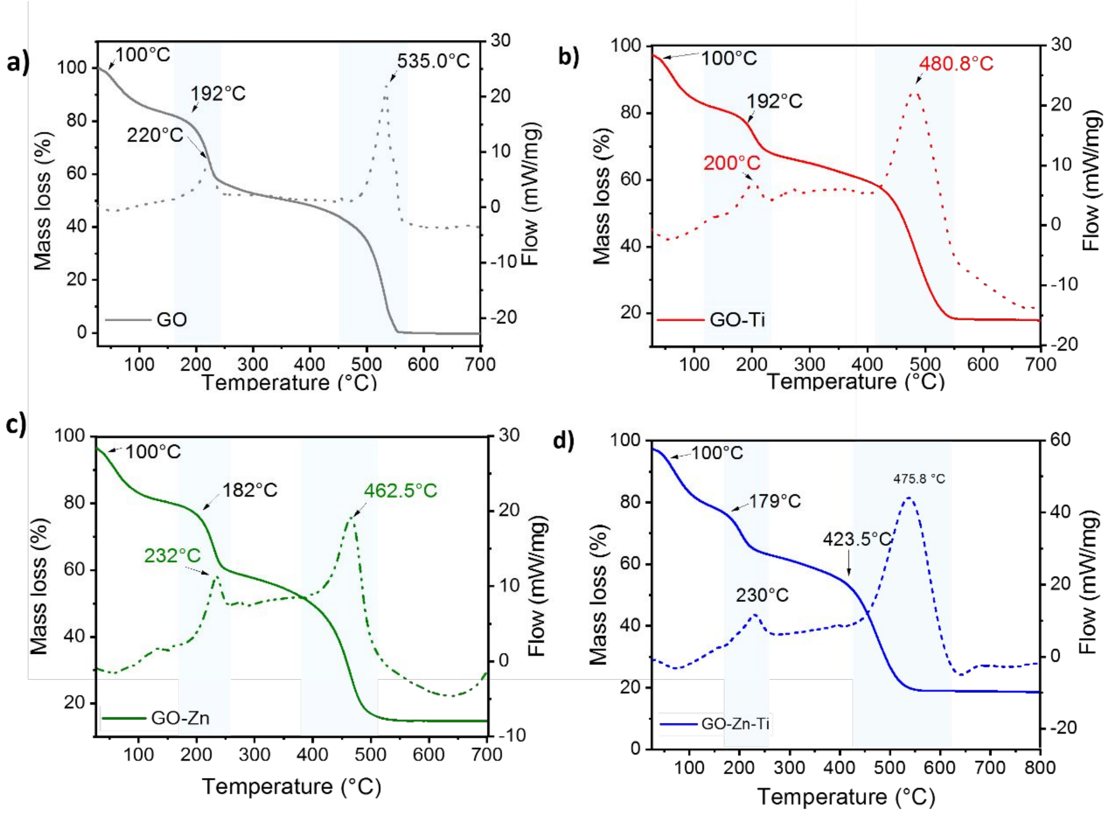

Figure S2. GO characterization by (a) MEV e EDS; (b) XRD; (c) Raman; XPS (d) survey and (e) high resolution C 1s; (f) FTIR.

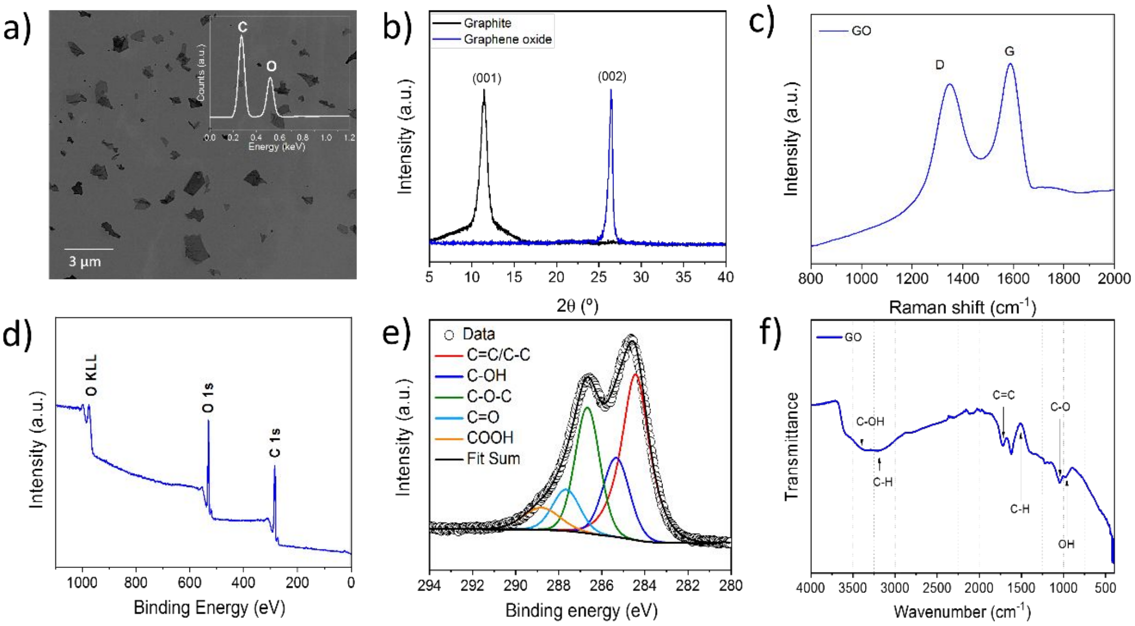

Figure S3. HRTEM image showing well defined lattice fringes with corresponding interplanar spacing determination for (a) TiO_2, (b) ZnO and (c) $Zn_2Ti_3O_8$.

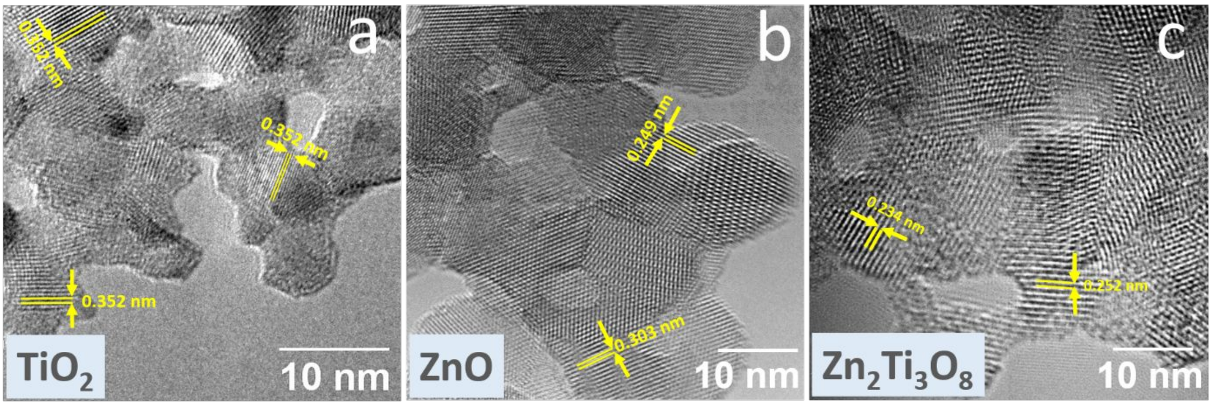

Table S1. Atomic percentages of the elements identified by X-ray photoelectron spectroscopy (XPS) survey spectra for the analyzed samples, obtained from quantitative surface composition analysis.

Sample	Element	Peak BE	Atomic %
	C1s	285.03	55.90
**ZnO**	O 1s	530.03	33.35
	Zn 2p	1021.53	10.74
	C1s	285.00	64.72
**TiO_2**	O 1s	529.50	28.53
	Ti 2p	458.50	6.75
	C 1s	284.55	51.28
**$Zn_2Ti_3O_8$**	O 1s	530.05	37.06
	Zn 2p	1021.55	4.34
	Ti 2p	458.55	7.28

Table S2. Electrochemical parameters derived from cyclic voltammograms recorded at different scan rates for GSM at the GCE-$Zn_2Ti_3O_8$, used to assess reversibility and charge transfer kinetics.

ν (V s^{-1})	E_{pa} (V)	I_{pa} (μA)	E_{pc} (V)	I_{pc} (μA)	I_{pa}/I_{pc}	E_{pa}-E_{pc} (V)
0.05	1.54	330.23	0.55	-123.00	2.68	0.99
0.10	1.58	577.18	0.59	-167.00	3.46	0.99
0.15	1.61	737.31	0.59	-213.00	3.46	1.02
0.20	1.64	855.41	0.59	-270.00	3.17	1.05
0.25	1.66	993.35	0.58	-329.00	3.02	1.08
0.30	1.66	1080.00	0.56	-380.00	2.84	1.10
0.35	1.68	1180.00	0.56	-414.00	2.85	1.12
0.40	1.70	1360.00	0.55	-511.00	2.66	1.15
0.45	1.72	1470.00	0.55	-531.00	2.77	1.17
0.50	1.73	1540.00	0.55	-553.00	2.78	1.18
0.55	1.73	1600.00	0.55	-573.00	2.79	1.18

Table S3. Recovery and precision results using the voltammetric method.

[GSM] Theoretical (μg/ml)	1st Analysis (A)	2nd Analysis (A)	3rd Analysis (A)	Average (A)	Standard deviation (A)	RSD (%)	[GSM] Measured (μg/ml)	Error (%)
0.4	22.2	21.9	21.0	21.7	0.6	2.9	0.4	119
0.7	26.6	25.5	26.2	26.1	0.5	2.1	0.7	95
1.1	34.6	33.5	35.5	34.5	1.0	2.9	1.0	95
1.5	43.3	41.8	43.8	43.0	1.1	2.5	1.4	100
1.8	52.7	51.6	52.7	52.3	0.6	1.2	1.8	100
2.2	62.5	60.3	62.6	61.8	1.3	2.1	2.2	99
2.6	71.9	70.1	70.4	70.8	1.0	1.4	2.6	98
2.9	79.5	78.5	79.9	79.3	0.7	0.9	3.0	98
3.3	87.5	86.5	86.7	86.9	0.6	0.6	3.3	99
3.6	93.3	92.6	93.1	93.0	0.4	0.4	3.6	98
